# Device-Measured 24-Hour Movement Behaviors and Blood Pressure: A 6-Part Compositional Individual Participant Data Analysis in the ProPASS Consortium

**DOI:** 10.1161/CIRCULATIONAHA.124.069820

**Published:** 2024-11-06

**Authors:** Joanna M. Blodgett, Matthew N. Ahmadi, Andrew J. Atkin, Richard M. Pulsford, Vegar Rangul, Sebastien Chastin, Hsiu-Wen Chan, Kristin Suorsa, Esmée A. Bakker, Nidhi Gupta, Pasan Hettiarachchi, Peter J. Johansson, Lauren B. Sherar, Borja del Pozo Cruz, Nicholas A. Koemel, Gita D. Mishra, Thijs M.H. Eijsvogels, Sari Stenholm, Alun D. Hughes, Armando Teixeira-Pinto, Ulf Ekelund, I-Min Lee, Andreas Holtermann, Annemarie Koster, Emmanuel Stamatakis, Mark Hamer

**Affiliations:** Institute of Sport Exercise and Health, Division of Surgery and Interventional Sciences (J.M.B., M.H.), University College London, United Kingdom.; University College London British Heart Foundation Research Accelerator (A.D.H.), University College London, United Kingdom.; University College London Hospitals, National Institute for Health and Care Research Biomedical Research Centre, United Kingdom (J.M.B., A.D.H., M.H.).; Mackenzie Wearables Research Hub, Charles Perkins Centre (M.N.A., N.A.K., E.S.), Faculty of Medicine and Health, University of Sydney, Australia.; School of Health Sciences (M.N.A., N.A.K., E.S.), Faculty of Medicine and Health, University of Sydney, Australia.; School of Public Health (A.T.-P.), Faculty of Medicine and Health, University of Sydney, Australia.; School of Health Sciences and Norwich Epidemiology Centre, University of East Anglia, United Kingdom (A.J.A.).; Faculty of Health and Life Sciences, University of Exeter, United Kingdom (R.M.P.).; HUNT Research Centre, Department of Public Health and Nursing, Faculty of Medicine and Health Sciences, Norwegian University of Science and Technology, Levanger (V.R.).; School of Health and Life Science, Glasgow Caledonian University, United Kingdom (S.C.).; Department of Movement and Sport Sciences, Ghent University, Belgium (S.C.).; School of Public Health, University of Queensland, Brisbane, Australia (H.-W.C., G.D.M.).; Department of Public Health (K.S., S.S.), University of Turku and Turku University Hospital, Finland.; Centre for Population Health Research (K.S., S.S.), University of Turku and Turku University Hospital, Finland.; Research Services (S.S.), University of Turku and Turku University Hospital, Finland.; Department of Physical Education and Sports, Faculty of Sport Sciences, Sport and Health University Research Institute, University of Granada, Spain (E.A.B., T.M.H.E.).; Department of Medical BioSciences, Exercise Physiology Research Group, Radboud University Medical Center, Nijmegen, The Netherlands (E.A.B.).; National Research Centre for the Working Environment, Copenhagen, Denmark (N.G., A.H.).; Occupational and Environmental Medicine (P.H., P.J.J.), Department of Medical Sciences, Uppsala University, Sweden.; Occupational and Environmental Medicine, Uppsala University Hospital, Sweden (P.J.J.).; School of Sport, Exercise and Health Sciences, Loughborough University, United Kingdom (L.B.S.).; Faculty of Sport Sciences, and Faculty of Biomedical and Health Sciences, Universidad Europea de Madrid, Madrid, Spain (B.d.P.C.).; Department of Sports Science and Clinical Biomechanics, Faculty of Health, Southern Denmark University, Odense, Denmark (B.d.P.C., A.H.).; Department of Population Science & Experimental Medicine, UCL Institute of Cardiovascular Science, UCL, United Kingdom (A.D.H.).; Department of Sport Medicine, Norwegian School of Sport Sciences, Oslo (U.E.).; Department of Chronic Diseases, Norwegian Public Health Institute, Oslo (U.E.).; Division of Preventive Medicine, Brigham and Women’s Hospital and Harvard Medical School, Boston, MA (I.M.L.).; Department of Epidemiology, Harvard T. H. Chan School of Public Health, Boston, MA (I.M.L.).; Maastricht University CAPRHI Care and Public Health Research Institute, Department of Social Medicine Maastricht, The Netherlands (A.K.).

**Keywords:** cardiometabolic risk factors, epidemiology, exercise, observational study, sedentary behavior, sleep, walking

## Abstract

**BACKGROUND:**

Blood pressure (BP)–lowering effects of structured exercise are well-established. Effects of 24-hour movement behaviors captured in free-living settings have received less attention. This cross-sectional study investigated associations between a 24-hour behavior composition comprising 6 parts (sleeping, sedentary behavior, standing, slow walking, fast walking, and combined exercise-like activity [eg, running and cycling]) and systolic BP (SBP) and diastolic BP (DBP).

**METHODS:**

Data from thigh-worn accelerometers and BP measurements were collected from 6 cohorts in the Prospective Physical Activity, Sitting and Sleep consortium (ProPASS) (n=14 761; mean±SD, 54.2±9.6 years). Individual participant analysis using compositional data analysis was conducted with adjustments for relevant harmonized covariates. Based on the average sample composition, reallocation plots examined estimated BP reductions through behavioral replacement; the theoretical benefits of optimal (ie, clinically meaningful improvement in SBP [2 mm Hg] or DBP [1 mm Hg]) and minimal (ie, 5-minute reallocation) behavioral replacements were identified.

**RESULTS:**

The average 24-hour composition consisted of sleeping (7.13±1.19 hours), sedentary behavior (10.7±1.9 hours), standing (3.2±1.1 hours), slow walking (1.6±0.6 hours), fast walking (1.1±0.5 hours), and exercise-like activity (16.0±16.3 minutes). More time spent exercising or sleeping, relative to other behaviors, was associated with lower BP. An additional 5 minutes of exercise-like activity was associated with estimated reductions of –0.68 mm Hg (95% CI, –0.15, –1.21) SBP and –0.54 mm Hg (95% CI, –0.19, 0.89) DBP. Clinically meaningful improvements in SBP and DBP were estimated after 20 to 27 minutes and 10 to 15 minutes of reallocation of time in other behaviors into additional exercise. Although more time spent being sedentary was adversely associated with SBP and DBP, there was minimal impact of standing or walking.

**CONCLUSIONS:**

Study findings reiterate the importance of exercise for BP control, suggesting that small additional amounts of exercise are associated with lower BP in a free-living setting.

**Clinical Perspective**

**What Is New?**

- We examined associations between 6 device-measured movement behaviors (sleeping, sedentary time, standing, slow walking, fast walking, and exercise-like activity) and blood pressure.
- As little as 5 minutes per day of additional exercise-like activity replacing any behavior was associated with lower systolic blood pressure (–0.68 mm Hg [95% CI, –0.15 to –1.21]) and diastolic blood pressure (–0.54 mm Hg [95% CI, –0.19 to –0.89]).
- An estimated 2 mm Hg (95% CI, 1.3 to 2.7) improvement in systolic blood pressure was observed if exercise-like activity replaced 20 minutes of fast walking, ≈21 minutes of sedentary time, ≈22 minutes of standing, ≈26 minutes of slow walking, or ≈27 minutes of sleeping.
- An estimated 1 mm Hg (0.6 to 1.4) improvement in diastolic blood pressure was observed if exercise-like activity replaced ≈10 minutes of fast walking, ≈11 minutes of sedentary time, ≈13 minutes of sleeping, ≈14 minutes of slow walking, or ≈15 minutes of standing.

**What Are the Clinical Implications?**

- Small and feasible changes to habitual exercise levels are estimated to have meaningful benefits on blood pressure, which can contribute to a reduction in prevalence of hypertension.
- Exercise-induced reductions in blood pressure can reduce prevalence of cardiovascular outcomes by 7% to 28% at the population level.
- Interventions targeting both the individual and the population (eg, public health policies) should consider the wider construct of the 24-hour day and incidental exercise embedded in daily activities.

Hypertension, characterized by consistently elevated blood pressure (BP) levels, is a significant global health concern, prevalent in >1 billion people globally.^1^ Despite pharmaceutical advances, the prevalence of hypertension has remained stable over recent decades (32% and 34% in 1999 and 2019, respectively),^1^ with an estimated 7.7 million to 10.4 million annual deaths attributable to elevated BP.^2^ Given the high global burden,^1^ there is a need to identify population-level modifiable risk factors.

Exercise levels are strongly associated with lower BP, with causal evidence from reviews of randomized controlled trials indicating robust BP-lowering effects of exercise (systolic BP [SBP]/diastolic BP [DBP]>5/2.5mm Hg).^3,4^ Observational evidence from free-living physical activity (PA) has also demonstrated associations with BP,^5,6^ although it lacks sufficient evidence on the effects of daily PA patterns, including type, intensity, and volume, on BP. In addition to exercise or PA alone, 24-hour movement patterns have emerged as potentially critical determinants of cardiovascular health.^7,8^ Increasingly, across national guidelines^9^ and research settings,^10,11^ the 24-hour day has been conceptualized as having 4 distinct behaviors: sleep, sedentary behavior, light PA, and moderate-to-vigorous PA.

Advancements in technology and processing algorithms enable a unique opportunity to examine movement behaviors with greater detail than these 4 parts. For example, evidence has demonstrated health benefits yielded from vigorous intermittent activity embedded in daily activities,^12^ distinct dose-response associations by PA type,^13^ and meaningful differences in associations of standing and light PA with cardiovascular health.^8^

There remains conflicting evidence on associations of different movement types, walking cadences, sleep durations, or standing positions with BP. Furthermore, new research has sought to identify minimal and optimal durations of activities associated with better health outcomes.^14,15^ For example, prospective data from the UK Biobank suggest that 15.0 (95% CI, 14.3, 15.7) and 56.5 (95% CI, 55.4, 55.6) minutes per week of vigorous PA represent minimal and optimal doses to yield a decrease in cardiovascular disease (CVD) risk.^15^ Meta-analyses have also suggested a U-shaped association between sleep duration and hypertension risk, yet optimal sleep duration remains unclear, and other movement behaviors are rarely incorporated.^16,17^ However, these studies have consistently examined PA in isolation without considering time spent in other 24-hour movement behaviors.

Compositional data analysis approaches examine various daily configurations to explore how redistributing movement within a day might affect health outcomes.^18^ In addition to better understanding how 24-hour movement behaviors may contribute to BP, this approach is promising for the development of personalized approaches for movement recommendations, as it could provide multiple meaningful ways to change behaviors. Quantifying the behavioral changes required to observe clinically meaningful changes in BP is crucial to identify optimal changes individuals could make as well as to identify whether minimal levels of PA (as suggested previously) are sufficient to have BP-lowering effects.

The primary aim of this cross-sectional study was to examine how a 6-part 24-hour movement composition consisting of sleep, sedentary behavior, standing, slow walking, fast walking, and combined exercise-like activity (eg, running and cycling) is associated with SBP and DBP. In addition, we modeled how reallocating time from one behavior into another (across any pair of behaviors) was associated with changes in SBP and DBP.

## METHODS

### Consortium Sample and Harmonization Process

Cross-sectional data were pooled from 6 observational cohort studies: TMS (The Maastricht Study, The Netherlands; n=7515; 48.9%),^19^ BCS70 (the 1970 British Birth Cohort Study, United Kingdom; n=5250; 34.1%),^20^ ALSWH (Australian Longitudinal Study on Women’s Health, Australia; n=985; 6.4%),^21^ DPhacto (Danish Physical Activity Cohort With Objective Measurements, Denmark; n=835; 5.4%),^22^ NES (Nijmegen Exercise Study, The Netherlands; n=537; 3.5%),^23^ and FIREA (Finnish Retirement and Aging Study, Finland; n=254; 1.7%).^24^ These 6 studies comprise the first pooled resource from the Prospective Physical Activity, Sitting and Sleep consortium (ProPASS) collaboration, an international research collaboration platform of observational cohort studies with thigh-worn accelerometry (ie, wearable movement trackers).^25^ Ethics approvals and consent were obtained at the cohort level. Detailed study information is available in Table S1 and has been published previously.^25–30^ All covariate, outcome, and raw accelerometer data were harmonized and pooled in one data set at the University of Sydney, adhering to specific cohort requirements through signed data transfer agreements (Supplemental Text S1 provides an overview of the harmonization process). The data that support the findings of this study are available from the corresponding author upon reasonable request following cohort-specific regulations.

### Movement Behaviors

Movement behavior data were collected through 7-day, 24-hour thigh-worn accelerometer protocols using ActivPAL3/4 (BCS70, TMS, ALSWH, and NES), Axivity (FIREA), or ActiGraph devices (DPhacto). Across studies, participants were fitted with or given the accelerometer on the day of the main data collection (Table S1). Raw accelerometer data underwent centralized processing using ActiPASS v1.56, which is device-agnostic for thigh-worn accelerometers and has been validated across all brands included in these analyses.^26–30^ Briefly, 6 movement behaviors were classified: sleep, sedentary behavior, standing, slow walking (cadence <100 steps/min), fast walking (cadence ≥100 steps/min), and combined “exercise-like” activities. Exercise-like activities were combined because of relatively small amounts of time spent running, cycling, or inclined stepping across the sample. Further detail on derivation and validation of these behavior types has been described previously,^26–30^ with more detail provided in Supplemental Text S1. A total of 15 376 individuals met wear time criteria (≥20 hours of wear/d, ≥1 walking period detected, and >0 minutes of sleep)^8^ and had ≥1 valid weekday and weekend day, and were therefore eligible for analysis (see sample size derivation in Figure S1). Average daily minutes spent in each behavior were computed.

### Blood Pressure

BP measurements used automated BP monitors administered by a research nurse or study staff member (see Table S2 for complete description by cohort). All cohorts implemented a 5- to 10-minute period of rest before measurements were taken. Three cohorts used 3 measurements to derive SBP and DBP averages, and 3 took the average of 2 measurements. In some studies, an additional measurement was taken when differences between readings exceeded >10 mm Hg for SBP or >5 mm Hg for DBP and included in the calculation of the average.

When individuals were taking prescribed antihypertensive medications, adjustments were made to BP outcomes following standard practice (+10 mm Hg to SBP or DBP, with sensitivity analyses exploring adding +5 mm Hg and +15 mm Hg).^31,32^ Antihypertensive medication status was ascertained across all cohorts (Table S2). Four cohorts asked participants to either bring or describe all prescription medications, which were subsequently coded using Anatomical Therapeutic Chemical classification (any of C02) or British National Formulary edition 69 codes (any of 0201 through 0207). The other 2 cohorts self-reported whether they took antihypertensive medications over the last 3 or 12 months.

### Covariates

Covariates were selected a priori.^10,11^ Harmonized covariates collected across all cohorts included age (years), sex (categorized as male or female), smoking status (nonsmokers or current smokers), and alcohol consumption (tertiles based on self-reported weekly intake). In addition, a subset of cohorts gathered data on mobility limitations (4 cohorts; scored on a continuous scale from 0 to 100 using the Short Form-36 10-item physical function subscale, for which 0 represents severe mobility limitations, and 100 signifies no mobility issues), occupational class (5 cohorts: not working, low, intermediate, or high occupational class), and level of education (4 cohorts; educational attainment level ranging from none or lower than high school to university degree or higher). To avoid overadjustment, body mass index was not included in the main models because of its likely role as a partial mediator; however, it was included in sensitivity analyses (see below). Complete details about the collection and harmonization of covariates in each cohort can be found in Table S2.

### Statistical Analyses

We defined a composition as the combined proportion of daily time spent in each of the 6 behaviors: sleep, sedentary behavior, standing, slow walking, fast walking, and combined exercise-like activity. First, we normalized average daily durations to ensure the collective sum equaled 1440 minutes (24 hours), accommodating any unrecorded periods. This 24-hour composition was then represented through a set of 5 isometric log-ratio (*ilr*) coordinates^18^ that capture the variability and magnitude of the relative time spent in each of the 6 behaviors. Each *ilr* coordinate within the set describes a specific behavior against the remaining behaviors. For example, the first coordinate describes time spent in the behavior of interest relative to time spent in the other 5 behaviors, the second coordinate describes time spent in the second behavior relative to time spent in the other 4, the third coordinate describes time spent in the third behavior relative to the other 3, etc. Integrating all 5 coordinates within a single regression model allows for the time spent in each of the behaviors relative to the others to be captured. Data were pivoted to create 6 distinct sets, each enabling the exploration of a single behavior relative to the other 5. For instance, the first pivoted set examined sleep compared with sedentary behavior, standing, slow walking, fast walking, and exercise. Any values of 0 were replaced with 1 second to allow derivation of coordinates (n=2 with no exercise-like activity).

We conducted a one-stage individual participant analysis using linear regressions to examine associations of each behavior (relative to others) with each BP outcome, repeating the below models for each set of pivoted coordinates. In initial regressions, we tested for sex interactions before building models in 2 stages: (1) adjusted for sex, age, and cohort; and (2) adjusted for sex, age, cohort, smoking, and alcohol. We repeated the models with additional adjustments for education, mobility limitations, and occupational class in cohorts with available data (ALSWH, BCS70, and TMS). Coefficients indicate the change in BP (mm Hg) for each 1-unit *ilr* increase. This coefficient is not directly interpretable; therefore, isotemporal substitution models were used to estimate how reallocation of time between behaviors may affect BP.^18^

Model reallocation plots were created using the sex–age cohort–adjusted models to maximize sample size and statistical power. Minimal daily reallocation times between pairs of behaviors to observe clinically relevant changes in BP (–1 mm Hg DBP and –2 mm Hg SBP) were identified,^33–36^ and differences in BP resulting from a 5-minute per day change between pairs of behavior (used previously as the minimum PA required for CVD benefits^15^) were also described when significant. Three primary samples were explored in this study: (1) those with complete data on 24-hour movement behaviors, SBP, and DBP (maximal sample; n=14 761); (2) those with complete data on main covariates (n=12 651); and (3) those with complete data on main and additional covariates (n=9799; 3 cohorts).

We conducted several sensitivity and subgroup analyses. First, we repeated the main models using 2 different adjustment factors for antihypertensive medication use (5 mm Hg and 15 mm Hg).^31,32^ Next, we repeated the above models in 2 subsamples: (1) those with no history of CVD (eg, heart disease, myocardial infarction, angina, stroke, etc; see Table S2 for description by cohort); and (2) those not currently on antihypertensive medications. Additional models also considered adjustment for body mass index in addition to the 2 primary adjustment models described above.

When sex interactions were evident across behaviors and BP outcomes, reallocation figures were stratified by sex. We conducted further subgroup analysis by repeating the main age, sex, and cohort-adjusted model in groups stratified by high and low groups (high: ≥median; low: <median) for each of sleep, sedentary behavior, and exercise-like activities. To formally test for differences, we tested interactions between the first *ilr* coordinate (eg, one behavior relative to the other 5) and a binary indicator of each high-low group. Finally, we stratified by cohort and repeated the main age- and sex-adjusted model to provide estimates by cohort as well as aggregate estimates from the 2-stage random-effects meta-regression.

All analyses were performed in RStudio 4.2.3 using the tidyverse, compositions, robCompositions, metafor, and zCompositions packages.

## RESULTS

### Sample Characteristics

Of 15 376 individuals with valid movement behavior data, 14 761 individuals had sufficient outcome data for inclusion in analyses (Figure S1). The sample spent most of their day sedentary (mean, 10.7±1.9 hours), with an average of 3.2±1.1 hours standing. Average time spent walking was similar at a slow (1.6±0.6 hours) and a fast pace (1.1±0.5 hours); finally, time spent engaging in combined exercise-like activities was the least frequent behavior (16.0±16.3 minutes). Average SBP and DBP were 132.2±19.1 mm Hg and 79.1±11.6 mm Hg, respectively, with 24.0% (n=3344) of the sample currently on antihypertensive medications. Approximately half of the sample was female (n=7828; 53%), with a mean age of 54.2 ±9.6 years (range, 18–87). Further details of sample characteristics are provided in the Table, with cohort-stratified characteristics in Table S3.

**Table.**
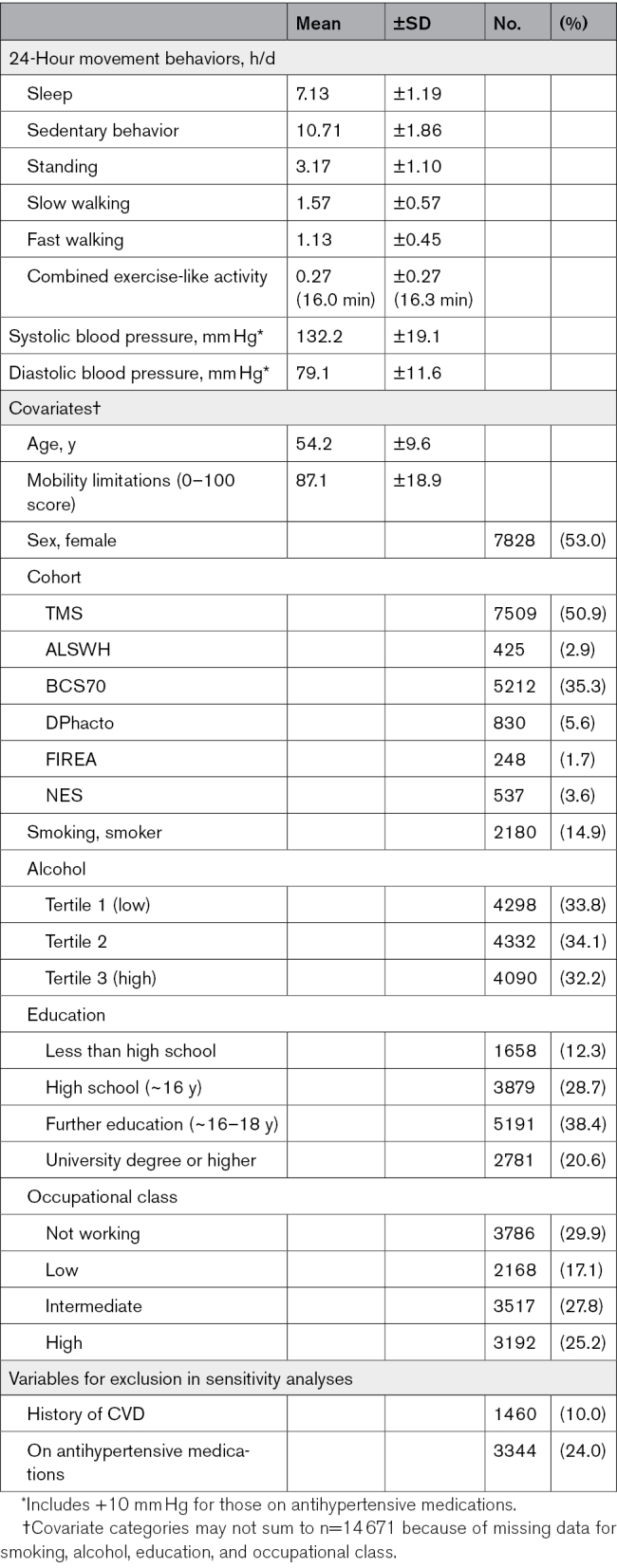
Characteristics of the Analytical Sample (n=14 761)

### Movement Behaviors and SBP

Because of minimal evidence of sex interactions, males and females were combined in models (see sensitivity analyses of sex differences below). Linear regressions of isometric log ratio coordinates (eg, one behavior relative to the other 5) demonstrated that, for the average sample composition, more time spent doing exercise-like activity or sleeping was associated with lower SBP, whereas more sedentary time was associated with higher SBP (Table S4). There was no association between time spent standing, slow walking, or fast walking, relative to other behaviors, and SBP. All associations remained after adjustment.

**Figure 1.**
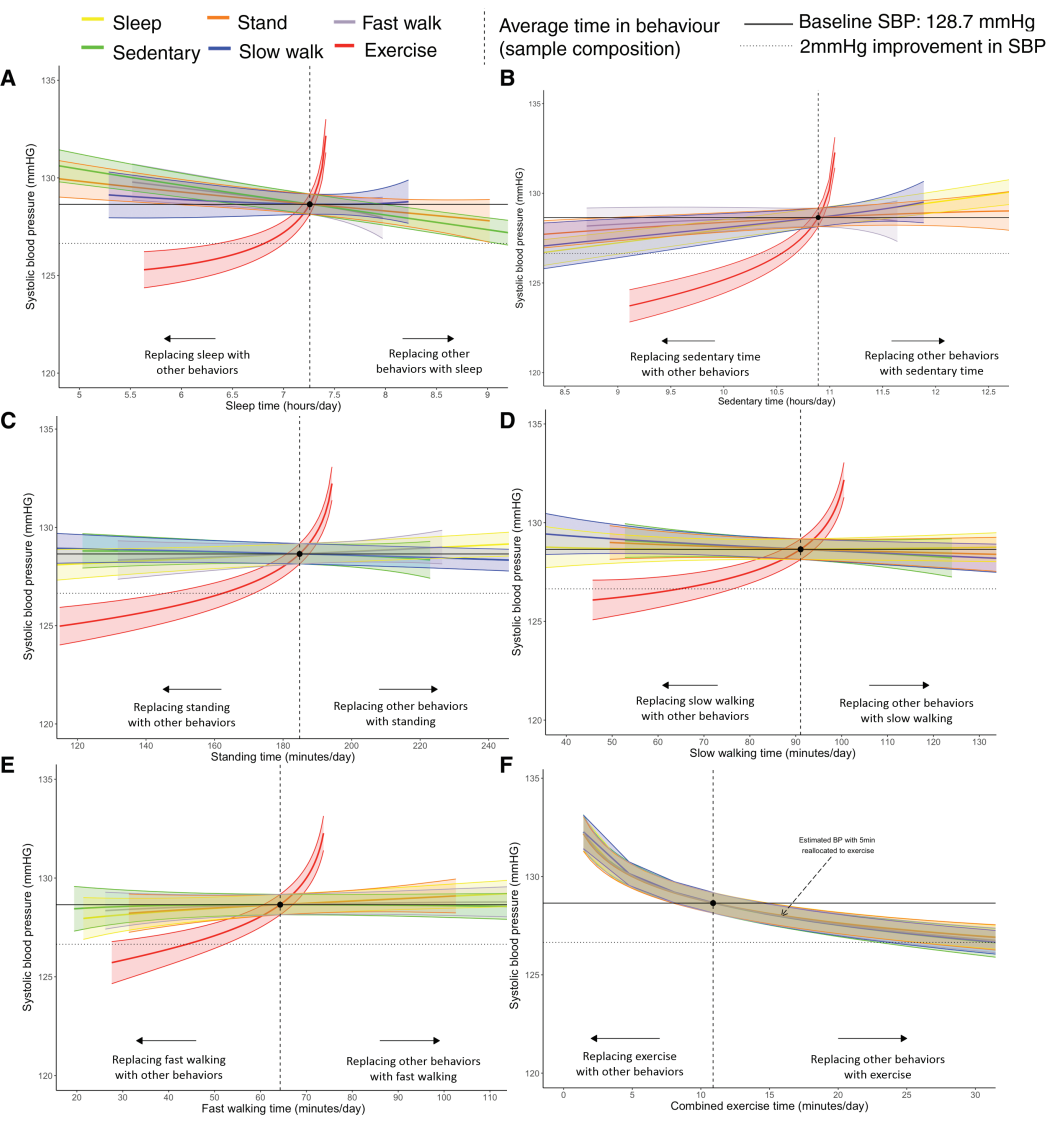
**Estimated change in systolic blood pressure (n=14 761) based on behavioral relocation from the average composition of the population**. Sleep (**A**), sedentary behavior (**B**), standing (**C**), slow walking (**D**), fast walking (**E**), and combined exercise-like activities (**F**). Data to the left of the reference line indicate the predicted change in systolic blood pressures if a given behavior were replaced by any of the other behaviors. Data to the right of the reference line indicate the predicted change if a given behavior replaced any of the other behaviors. Model adjusted for sex (reference: female), age (reference: 54.2 years; mean-centered), and cohort (reference: The Maastricht Study). Reallocations are based on baseline systolic blood pressure (SBP; 128.7 mm Hg) expected given the average sample composition (sleep, 7.3 hours; sedentary behavior, 10.9 hours; stand, 3.1 hours; slow walk, 1.5 hours; fast walk, 1.1 hours; and combined exercise-like, 10.9 minutes per day).

Reallocation plots indicated that replacing any behavior with exercise-like activity was associated with the strongest estimated reduction in SBP (Figure 1). Based on the average sample composition, statistically significant improvements in SBP were observed when an additional 5 minutes of any behavior was reallocated into exercise-like activity. Replacing 5 minutes of sedentary time with exercise-like activity demonstrated the largest improvement in SBP (–0.68; 95% CI, –0.15 to –1.21), with similar decreases observed when replacing standing, slow walking, fast walking, or sleep (Figure 1F). Five-minute reallocations between any other pair of behaviors did not result in statistically significant improvements in SBP.

When examining the behavior changes needed to yield clinically significant changes in SBP, exercise-like activity continued to be the most important. An estimated 2 mm Hg (95% CI, 1.3 to 2.7) improvement in SBP was observed if exercise-like activity replaced ≈20 minutes of fast walking, ≈21 minutes of sedentary time, ≈22 minutes of standing, ≈26 minutes of slow walking, or ≈27 minutes of sleeping while keeping other behaviors constant. Beyond exercise-like activity, a clinically significant 2 mm Hg improvement in SBP was only observed if ≈2 hours and 50 minutes of sedentary time was replaced by sleep. No reallocation between any amount of time spent standing, slow walking, or fast walking yielded a clinically significant change (Figure 1A).

### Movement Behaviors and DBP

More time spent in exercise-like activity or sleep was associated with lower DBP, whereas more time spent sedentary was associated with higher DBP (Table S5). There was evidence to suggest that more time spent standing and less time spent walking fast were associated with lower DBP; however, associations for fast walking attenuated after adjustment for covariates. There was no association between slow walking and DBP.

**Figure 2.**
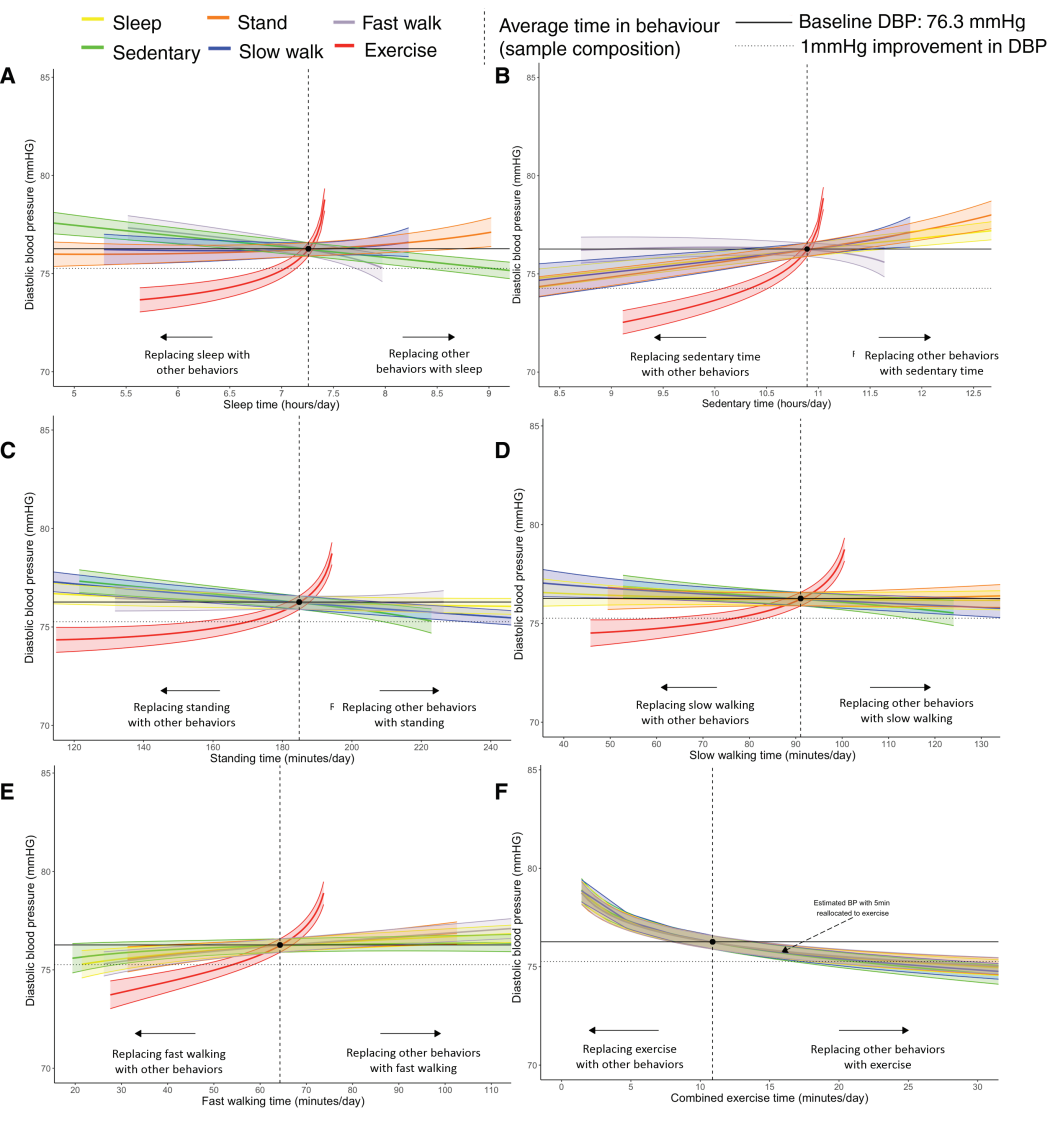
**Theoretical change in diastolic blood pressure (n=14 761) based on behavioral relocation from the average behavior composition of the population.** Sleep (**A**), sedentary behavior (**B**), standing (**C**), slow walking (**D**), fast walking (**E**), and combined exercise-like activity (**F**). Data to the left of the reference line indicate the predicted change in systolic blood pressures if a given behavior is replaced by any of the other behaviors. Data to the right of the reference line indicate the predicted change if a given behavior replaces any of the other behaviors. Model adjusted for sex (reference: female), age (reference: 54.2 years; mean-centered), and cohort (reference: The Maastricht Study). Reallocations are based on baseline diastolic blood pressure (DBP; 76.25 mm Hg) expected given the average sample composition (sleep, 7.3 hours; sedentary behavior, 10.9 hours; stand, 3.1 hours; slow walk, 1.5 hours; fast walk, 1.1 hours; combined exercise-like, 10.9 minutes per day).

These associations were reflected in the reallocation plots, in which replacement of any behavior with exercise-like activity had the strongest estimated reductions in DBP (Figure 2). Reallocation of 5 minutes into exercise-like activity demonstrated comparable changes in DBP regardless of the type of behavior it replaced; for example, replacing an additional 5 minutes of sedentary time with exercise-like activity equated to a –0.54 mm Hg (95% CI, –0.19 to –0.89) improvement in DBP (Figure 2F). No 5-minute reallocations between any other pair of behaviors resulted in any statistically significant improvement in DBP.

Clinically significant improvements of 1 mm Hg (95% CI, 0.6 to 1.4) in DBP were estimated if exercise-like activity replaced an additional ≈10 minutes of fast walking, ≈11 minutes of sedentary time, ≈13 minutes of sleeping, ≈14 minutes of slow walking, or ≈15 minutes standing. Separate from exercise-like activity, a 1 mm Hg lower DBP was possible through replacing sedentary time with standing, slow walking, or sleeping; however, substantial reductions in sedentary time beyond the baseline average composition were required to observe such a change (eg, 78 minutes, 95 minutes, and 106 minutes, respectively). No other displacement between behaviors could yield a 1 mm Hg change.

### Sensitivity Analyses

SBP associations did not change when the antihypertensive medication adjustment was changed to 5 mm Hg or 15 mm Hg (Table S6), nor when analyses were repeated in subsamples excluding those on antihypertensive medications or those with a history of CVD (Table S7). However, there was evidence that more time spent walking fast, relative to other behaviors, was associated with worse DBP when DBP was adjusted by 5 mm Hg (Table S8) or when those on antihypertensive medications were excluded (Table S9). Positive associations between more time spent slow walking and lower DBP emerged in analyses that excluded those on antihypertensive medications. Finally, additionally adjusting for body mass index resulted in some attenuation of estimates; more time spent sedentary remained associated with greater DBP but not SBP (Table S10).

### Subgroup Analyses

As described above, there was minimal evidence of sex interactions between *ilr* coordinates and SBP or DBP, with no significant interactions for DBP and just 2 significant interactions for SBP. These interaction terms suggested that the positive association of sleep and the negative association of sedentary behavior with SBP were slightly stronger in females (Figure S2).

Behavior-stratified estimates of *ilr* coordinates and BP associations are provided in Table S11: high sleep (≥7.2 h/d; n=7381), low sleep (<7.2 h/d; n=7380), high sedentary behavior (≥10.7 h/d; n=7381), low sedentary behavior (<10.7 h/d; n=7380), high exercise (≥10.3 min/d; n=7381), and low exercise (<10.3 min/d; n=7380). Briefly, there were no interactions between sedentary behavior or exercise levels with SBP. In contrast to the main models, there was no association between either sleep or sedentary behavior, relative to other behaviors, and SBP in those with high sleep levels (≥7.2 h/d). However, in those with insufficient sleep (<7.2 h/d), there remained strong associations between more sleep and less sedentary behavior with lower SBP.

Subgroup differences for DBP were in the same direction as in SBP, but more prominent (Table S11). For example, positive associations between more time spent exercising and lower DBP were stronger in those with low sedentary time (<10.7 h/d) and those with high sleep levels (**≥**7.2 h/d). The beneficial association of more time sleeping with lower DBP was not observed in those with low sedentary time, whereas more sleep was detrimental in those with high sleep levels. Adverse associations between more time spent sedentary and higher DBP were strongest in those with insufficient sleep and those with high levels of sedentary behavior. Last, there was evidence to suggest that more time spent standing could be beneficial for DBP in those with high exercise levels (≥10.3 min/d); conversely, more time spent walking slowly was associated with lower DBP in those with low exercise levels (<10.3 min/d) only.

Characteristics of the 3 primary samples (differing because of missing data) as well as those excluded from each analysis are provided in Table S12. Those excluded from all samples tended to be younger and were more likely to be females. Briefly, those excluded from the first 2 samples (because of missing SBP, DBP, smoking, or alcohol use) spent less time doing exercise-like activities or sitting, but more time lying or sleeping. In the 2-stage meta-analysis, associations between all behaviors and both BP outcomes did not change, with low *I*^2^ heterogeneity for sleep and sedentary behavior and moderate-high heterogeneity for other behaviors (Figures S3 and S4). There were some differences in compositional associations by cohort; most notably, some cohorts (DPhacto and ALSWH) demonstrated favorable associations between slow and fast walking and lower BP, whereas there were no associations between exercise-like activities and BP in the DPhacto cohort.

## DISCUSSION

We conducted cross-sectional compositional data analyses to explore novel associations between BP and 6 daily movement behaviors: sleeping, sedentary behavior, standing, slow walking, fast walking, and exercise-like activity. Our findings corroborate the importance of BP-lowering effects of exercise-like activities, demonstrating that small amounts of additional time in exercise-like activities was associated with a reduction in BP, regardless of the behavior it replaced. For example, 5 minutes of additional exercise-like activity was associated with –0.68 and –0.54 mm Hg decreases in SBP and DBP, respectively. More substantial reallocation of sleeping, sedentary behavior, standing, or walking time into exercise-like activity were required to achieve clinically meaningful decreases in BP (SBP, 20–27 min/d; DBP, 10–15 min/d). Although our findings suggest adverse effects of prolonged sedentary time and a positive impact of more sleep on BP, the magnitude of reallocation into and out of these behaviors required for clinically meaningful improvements in BP may not be achievable for many. Findings emphasize the potential of small amounts of daily exercise-like activity to aid BP management at both individual and population levels, while providing a more tentative perspective of the impact of altering sleeping, standing, walking, or sedentary behaviors for optimizing BP.

### Exercise and BP

Our results align with well-established evidence on the antihypertensive effects of exercise.^3,4^ However, we provide novel evidence on “minimal” and “optimal” levels of exercise-like activity to improve BP in the context of 24-hour movement. Recent UK Biobank evidence has highlighted smaller minimum (15 min/week or 2.1 min/d) and optimal (56.5 min/week or 8.1 min/d) amounts of vigorous PA for reduced CVD.^15^ We report that 5 minutes per day of minimal increase in exercise-like activities was associated with significantly lower SBP (–0.68 [95% CI, 0.15 to –1.21]) and DBP (–0.54 [95% CI, –0.19 to 0.89]) regardless of the behavior replaced, with 10 to 27 minutes per day required for clinically meaningful improvements (DBP, 10–15 min/d; SBP, 20–27 min/d). The exercise-like activities modeled in our study encompassed activities such as running, cycling, or inclined walking, and could include both structured, intentional exercise and incidental daily activities such as running for a bus or climbing stairs.

A meta-analysis of 93 exercise trials (≥4 weeks) suggested that optimal BP-lowering effects emerged at 2.5 to 3.5 hours week of moderate-vigorous dynamic endurance training (≈30–45 min/session); this equated to 3 to 5 mm Hg improvements in SBP and 2 to 4 mm Hg improvements in DBP.^37^ This offers an interesting comparison to the 20 to 27 minutes (2.3–3.2 h/week) and 10 to 15 minutes (1.2–1.75 h/week) of additional daily exercise-like activity required for clinically meaningful improvements in SBP and DBP, respectively. Despite the known benefits of exercise, participation rates in structured exercise sessions remain low because of poor feasibility and desire for many middle-aged adults.^38,39^ Therefore, an important next step is to examine comparability of benefits yielded from structured exercise and incidental high-intensity activity, which are both captured in the free-living protocol in this study. For example, “vigorous intermittent lifestyle physical activity” (VILPA)^14^ is defined as short (typically up to 1 or 2 minutes) intermittent bouts of incidental, higher-intensity activity that happen during typical daily activities. Evidence from non-exercisers in the UK Biobank suggests that as few as 3 bouts per day of VILPA (lasting ≈1 or 2 minutes each) was associated with a 48% to 49% reduction in CVD risk^12^; to our knowledge, associations between VILPA and BP have not yet been explored, although BP may be one intermediate pathway through which VILPA or exercise-like activities may reduce overall CVD risk.

The strength of the findings for combined exercise-like activity compared with other 24-hour behaviors highlights the necessity for higher intensity activities that challenge the cardiovascular system. This is consistent with recent evidence demonstrating that short activity bursts that included vigorous bouts lowered CVD risk, but short bursts without vigorous activity did not.^40^ Acute physiological mechanisms include vasodilation through production of nitric oxide in the endothelium and reduced arterial stiffness through production of elastin and collagen, whereas long-term benefits may involve mediators such as adiposity loss and other metabolic improvements.^41^ PA conducted at low intensity (eg, light walking or ambulatory movement) may be insufficient to yield these physiological adaptations, especially at low to moderate volumes. A recent meta-analysis of 270 exercise randomized controlled trials concluded that walking was the least effective intervention for lowering BP compared with exercise interventions such as cycling, running, strength training, aerobic training, and interval training.^3^ It was notable that subgroups with healthier behaviors (eg, higher sleep or lower sedentary time) had stronger associations between exercise time and BP. This further highlights the interdependence of the 24-hour movement behaviors and is consistent with evidence suggesting that sleep deprivation may reduce exercise benefits.^42^

### Standing, Walking, and BP

Beyond the benefits attributed to exercise, substantial replacement of sedentary time with standing or slow walking were required to observe any clinically meaningful change in DBP (78 min/d and 95 min/d, respectively), with no possible reallocation yielding a clinically significant change in SBP. Benefits of walking for cardiovascular health are highly dependent on individual-specific intensity and baseline health; this supports evidence suggesting that walkingm after higher intensity activity is accounted for, may be insufficient to induce meaningful changes in the BP of healthy individuals.^41^ The results reported here are consistent with the aforementioned systematic review highlighting the comparative inferiority of walking interventions on BP^3^ but contrast evidence from another review suggesting that walking interventions can reduce BP across all sexes and ages.^43^ Limitations of interventions are the inability to capture vigorous incidental PA or structured exercise time outside of the walking intervention.^43^

Finally, benefits of walking for lower BP emerged in individuals with low exercise levels (<10.3 min/d), and in the DPhacto cohort, a sample of primarily “blue-collar” workers from the manufacturing, transportation, and cleaning sectors.^22^ There were no benefits of exercise-like activity on either BP outcome in DPhacto. These subgroup findings provide insight into how PA benefits may differ because of individual circumstances such as a physically demanding occupation or inability or unwillingness to participate in exercise-like activities.^44^ Further explorations into how walking intensity may contribute to BP management, differ by occupation or baseline 24-hour profile, and contribute to meaningful changes in cardiovascular health through non-BP mechanisms are important next steps.

### Sleep and BP

The benefits of longer sleep duration for lower BP may be related to restorative processes that occur while sleeping.^45^ During sleep, there is reduced sympathetic activity; for example, nocturnal dipping is a common physiological occurrence, with a habitual reduction of 10% to 20% in BP compared with wakefulness.^45^ Over time, chronic sleep deprivation could lead to systemic arterial hypertension through increased cardiovascular strain, vasoconstriction, and sympathetic nervous system activity.^46^ Therefore, it is unsurprising that increasing sleep, if high-quality, at the expense of sedentary or light PA could reduce BP by providing increased opportunities for restorative processes that reduce sympathetic activity (ie, vasodilation or decreased pressure) and alleviate strain on the cardiovascular system. However, a substantial amount of time reallocated from sedentary behavior to sleep (eg, 2 hours and 50 minutes for SBP, 1 hour and 46 minutes for DBP) was required to yield clinically meaningful reductions in BP. Subgroup analysis suggested that more sleep was detrimental for BP for those already getting high levels of sleep; this is consistent with evidence on adverse effects of long sleeping durations on high BP.^16,17^ Other subgroup differences highlighted interactions between sleep and other behaviors. For example, positive associations of sleep with lower BP were stronger for those with high levels of sedentary behavior and weaker for those with sufficiently low sedentary behavior.

### Implications

Implementing daily changes of an additional 5 minutes of exercise-like activities provides realistic behavioral changes that could be readily integrated into daily habits and activities. To yield clinically meaningful reductions in BP, exercise-like activities may need to replace ≈10 to 30 minutes of time spent in other behaviors. This is comparable to optimal doses of structured exercise identified in exercise trial studies.^37^ Behavioral changes below the optimal time allocations required for the clinically meaningful improvement in 2 mm Hg in SBP or 1 mm Hg in DBP may still have meaningful impacts. A 2 mm Hg reduction in SBP is not negligible.^33–36,47^ Commonly cited evidence from a *Lancet* study of >1 million individuals indicated that a 2 mm Hg reduction in SBP translates to a 7% to 10% reduction in ischemic heart disease and stroke mortality.^34^ Similarly, a 1 mm Hg reduction in DBP is associated with ≈10% reductions in prevalence of CVD events including heart failure, stroke, major cardiovascular events, and cardiovascular death.^47^ Although the primary compositional results were consistent across additional analyses, subgroup results highlight the need to consider individual 24-hour movement profiles when providing guidance on changes to activity, sedentary time, and sleep.

### Strengths and Limitations

We used data from ≈15 000 individuals from 6 cohort studies across 5 countries, increasing the generalizability of our findings. The use of thigh-worn accelerometers, the preferred device placement for detecting posture, and the uniform processing of raw accelerometer data using the ActiPASS software enabled us to differentiate between distinct movement behavior types, resulting in a 6-part composition providing finer granularity on how each behavior, relative to others, is associated with BP. BP was objectively measured by each cohort using standardized protocols, with minimal missing data. Our results were robust across substantial additional analyses, including varying the BP adjustment factor for antihypertensive medication use, excluding those on antihypertensive medications or with a history of CVD and considering different subgroups by sex and individual behavior.

There were some limitations. First, we are unable to infer causal associations because of the cross-sectional data and the modeling analyses that estimated theoretical improvements in BP resulting from reallocation between pairs of behaviors. Misclassification, overlap, or undetected measurement of some behaviors is likely. For example, static resistance training exercise would be classified as either sedentary time (ie, sitting or lying) or standing because of the posture of the position, whereas swimming is not recognized by the algorithm and thus would have been categorized as “other” and excluded from the composition. Inferences about activity intensity can be made about the exercise-like activities because of expected higher intensities of cycling, running, and inclined walking; however, movement type and not the intensity were used to identify these behaviors. We were unable to capture sleep quality, which may have stronger associations with BP than sleep duration alone.^48^ Despite including participants from 5 countries across 3 continents, the pooled sample lacked ethnic and racial diversity. As ProPASS expands to other cohort studies^49^ (eg, developing prospective partnerships with organizations and cohorts in low and middle income countries)^50^ the global representativeness of the consortium will improve. Finally, we did not explore how duration of exercise bouts (eg, structured bouts versus incidental activity across the week) may have influenced associations because only average daily time spent in exercise-like activities was included in the composition.

### Perspectives

Our findings reinforce that in free-living environments, more time spent in exercise-like activities has the strongest association with BP, and even small changes to daily movement patterns can elicit clinically meaningful improvements. Although evidence for the positive impact of sleep on BP was observed, the volume of reallocation from other behaviors to yield meaningful changes in BP may not be feasible for many. Benefits of replacing one behavior with another may differ by sex or baseline 24-hour movement profile (eg, low or high exercise, sleep, or sedentary behavior levels). Future work must examine longitudinal associations between movement compositions and cardiometabolic outcomes, better explore interindividual differences in reallocation, and explore how different movement bouts (duration, frequency, and intensity) and variability in patterns of sleep, sedentary behavior, or activity accumulation across the day and week may contribute to reducing risk of hypertension. Overall, our findings underscore the irreplaceable role that exercise-like activities may have in yielding benefits in BP management.

## ARTICLE INFORMATION

### Acknowledgments

The data on which this research is based were drawn from 6 observational studies in the Netherlands, United Kingdom, Australia, Denmark, and Finland. We are grateful to all participants who provided survey data.

### Sources of Funding

This project was funded by a British Heart Foundation special grant (SP/F/20/150002) and National Health and Medical Research Council (Australia) investigator (APP1194510) and ideas (APP1180812) grants. The establishment of the ProPASS consortium was supported by an unrestricted 2018-20 grant by PAL Technologies (Glasgow, United Kingdom). ActiPASS development was partly funded by FORTE, Swedish Research Council for Health, Working Life and Welfare (2021-01561). M.N.A. is supported by the National Heart Foundation (APP 107158). E.S. is funded by a National Health and Medical Research Council Investigator Grant (APP1194510). G.D.M. is supported by a National Health and Medical Research Council principal research fellowship (APP1121844). A.D.H. receives support from the British Heart Foundation, the Horizon 2020 Framework and the Horizon Europe Programme of the European Union, the National Institute for Health Research University College London Hospitals Biomedical Research Centre, the United Kingdom Medical Research Council, the National Institute for Health Research, and the Wellcome Trust, and works in a unit that receives support from the United Kingdom Medical Research Council. E.A.B. has received funding from the European Union Horizon 2020 research and innovation program under the Marie Sklodowska-Curie grant agreement (No. 101064851).

### Disclosures

E.S. is a paid consultant and holds equity in Complement Theory Inc, a US-based startup company for which products and services relate to the contents of this article. The other authors report no conflicts.

### Supplemental Material

Supplemental Text S1

Figures S1–S4

Tables S1–S12

## Appendix

ProPASS Collaboration: Hans Savelberg, PhD; Bastiaan de Galan, PhD; Carla van de Kallen, PhD; Dick H.J. Thijssen, PhD

## Supplementary Material


