# Supplementary material for: Device-Measured 24-Hour Movement Behaviors and Blood Pressure: A 6-Part Compositional Individual Participant Data Analysis in the ProPASS Consortium
Source: Circulation. 2024 Nov 6;151(2):159–70. doi: 10.1161/CIRCULATIONAHA.124.069820 (PMC11732261; doi:10.1161/CIRCULATIONAHA.124.069820)

SUPPLEMENTAL MATERIAL

Supplemental Methods S1. Additional methodological information on harmonisation of non-accelerometer across cohort and derivation of behaviors included in the 6-part composition

Supplemental Methods S2. Details of individual cohort funding

Table S1. Overview of individual cohort details

Table S2. Assessment and harmonisation of blood pressure and covariate measures by cohort

Table S3. Cohort-stratified means for 24-hour movement behaviors and BP outcomes

Table S4. Estimated regression coefficients [a] of isometric log ratios of each behavior (ILR coordinate representing 1 behavior relative to other 6) and **systolic blood pressure**

Table S5. Estimated regression coefficients[a] of isometric log ratios of each behavior (ILR coordinate representing 1 behavior relative to other 6) and **diastolic blood pressure**

Table S6. Estimated regression coefficients[a] of isometric log ratios of each behavior (ILR coordinate representing one behavior relative to other five) and **systolic blood pressure** with **5mmHg and 15mmHg transformation** for those currently on anti-hypertensive medication (per Tobin et al., 2005)

Table S7. Estimated regression coefficients[a] of isometric log ratios of each behavior (ILR coordinate representing 1 behavior relative to other 6) and **systolic blood pressure** in subsamples of those **not on anti-hypertensive medications** and those **without history of cardiovascular disease**

Table S8. Estimated regression coefficients[a] of isometric log ratios of each behavior (ILR coordinate representing 1 behavior relative to other 6) and **diastolic blood pressure** with **5mmHg and 15mmHg transformation** for those currently on anti-hypertensive medication (per Tobin et al., 2005)

Table S9. Estimated regression coefficients[a] of isometric log ratios of each behavior (ILR coordinate representing 1 behavior relative to other 6) and **diastolic blood pressure** in subsamples of those **not on anti-hypertensive medications** and those **without history of cardiovascular disease**

Table S10. Estimated regression coefficients[a] of isometric log ratios of each behavior (ILR coordinate representing 1 behavior relative to other 6) and **systolic & diastolic blood pressure,** with additional a**djustment for BMI**

Table S11. Estimated regression coefficients[a] of isometric log ratios of each behavior (ILR coordinate representing 1 behavior relative to other 6) and each of **systolic and diastolic blood pressure** stratified by **subgroups of sleep, sedentary behavior and exercise** (based on median cut-points)

Table S12. Characteristics of analytical samples and those excluded due to missing data

Figure S1. Derivation of analytical sample size. See cohort profiles for more information on rates of participation in accelerometer data collection and reasons for missing data

Figure S2. Theoretical change in **systolic** blood pressure (n=) based on behavioral relocation for (*A*) sleep in females; (*B*) sleep in males; (C) sitting in females; (D) sitting in males. Data to the left of the reference line indicate the predicted change in systolic blood pressures if a given behavior is replaced by any of the other behaviors. Data to the right of the reference line indicate the predicted change if a given behavior replaces any of the other behaviors. Model adjusted for age (ref: 54.2 years; mean-centred), and cohort (ref: Maastricht Study).

Figure S3. Forest plot of two-stage meta-analysis demonstrating estimates by cohort for **systolic blood pressure**

Figure S4. Forest plot of two-stage meta-analysis demonstrating estimates by cohort for **diastolic blood pressure**

Supplemental M1. Additional methodological information on harmonisation of non-accelerometer across cohort and derivation of behaviors included in the 6-part composition

Harmonisation of non-accelerometer data:
Based on an initial list of 30 core non-accelerometer related variables (including cardiometabolic outcomes and relevant covariates), harmonized variables were derived using an iterative process from a team of harmonization panel members and experts from the ProPASS consortium. Continuous variables were considered harmonizable if they could be transformed to comparable units (e.g. years for age, mmHg for BP). Categorical variables were compared across each cohort and where feasible, combined categories were derived to maximise the number of cohorts with harmonizable data. A comprehensive data dictionary was developed and all original cohort data were recoded to derive the ProPASS harmonized variables for analysis (see Supplementary Table 2 & 3 for cohort-specific description of outcome and covariate data). Coding of all variables across each cohort was confirmed by a second member of the harmonization panel.

Overview of ActiPASS software:
ActiPASS is a MATLAB program that operates in 2-second windows with a 50% overlap, generating 1-second windows and employs algorithms for non-wear, sleep detection, posture, and activity intensity derived from cadence. It has high accuracy (>90%) in identifying activity types and sleep (80%) and for thigh-worn accelerometers, it is device agnostic and has been validated across all included accelerometer brands.

ActiPASS uses the Acti4 algorithm to identify the 24-hour movement behaviors. Briefly, physical behaviors are identified using information about the angle of the thigh-worn accelerometer relative to the line of gravity, combined with the standard deviation of accelerations in three directions. To differentiate between lying and sitting, information about rolling of the thigh is used. “Sleep” is identifying in a two-stage process. During the first stage, periods of likely times-in-bed are identified by considering long continuous periods of lying-down and also prolonged periods of sitting to a lesser extent. “Sleep” is then defined as the total time of sleep detected within such times-in-bed periods according to ActiPASS sleep algorithm. The accelerometery non-wear algorithm is based on absence of device movement and with raw accelerometry data; it is standard practice to use the standard deviation to detect periods of no device movement to identify non-wear episodes. This is not merely device agnostic for thigh-worn accelerometers, but it is also placement agnostic with studies using thigh, hip, wrist, ankle and lower back placements all using standard deviation (Alaqil et al., 2024 doi: 10.1186/s12889-024-18867-2.; Baldanzi et al., 2024 https://doi.org/10.1016/j.ebiom.2024.104989; Fairclough et al., 2016 doi: 10.1249/MSS.0000000000000771.; Khurshid et al., 2023 doi: 10.1001/jama.2023.10875.; Vert et al. 2022 doi: 10.1136/bmjopen-2014-007447 ; Zhou AM et al. doi: 10.1136/bmjopen-2014-007447).

The table below provides an explanation of the description of each movement behavior, an example, ActiPASS v1.56 variable names and the relevant references.

Behavior	Description of derivation of behavior	Example of activities	ActiPASS v1.56 variable names (or combined equation)	Relevant references for detailed description of processing method
Sleep	"Sleep" is identifying in a two-stage process. During the first stage, periods of likely times-in-bed are identified by considering long continuous periods of lying-down *(Hettiarcachchi et al., 2021)* and also prolonged periods of sitting to a lesser extent. "Sleep" is then defined as the total time of sleep detected within such times-in-bed periods according to ActiPASS sleep algorithm *(Johansson et al., 2023)*	Sleeping in a bed, sofa or lying very still in bed or in a sofa	Sleep	Hettiarachchi et al. https://doi.org/10.3390/s21030904<br><br>Johansson et al. https://doi.org/10.1111/jsr.13725
Sedentary behavior	"Sedentary behavior" is defined as:<br>1) Total time of sitting or lying according to Acti4/ActiPASS algorithm *(Skotte et al., 2014)* outside the periods flagged as sleep-interval(s).<br>**and**<br>2) The total non-sleeping time during sleep-interval(s)<br>Where a sleep- interval *(Ibáñez et al., 2018)* is defined as the time between the first sleep-onset and last sleep- offset during periods times-in-bed	Sitting and lying	SitLie + (SleepInterval - Sleep)	Skotte et al. https://doi.org/10.1123/jpah.2011-0347<br><br>Ibáñez et al. https://doi.org/10.7717/peerj.4849
Standing	"Standing" is defined as the total time of standing still according to Acti4/ActiPASS algorithm *(Skotte et al., 2014)*	Standing	Stand	Skotte et al. https://doi.org/10.1123/jpah.2011-0347

Slow walking	"Slow walking" is defined as:<br>1) time of walking according to ActiPASS/Acti4 algorithm outside periods flagged as sleep-interval(s) with a stepping cadence *(Ingebrigtsen et al., 2013)* below 100 steps/min *(Tudor-Locke et al., 2018)* outside periods flagged as sleep-interval(s)<br>**and**<br>2) time of upright posture without purposeful walking but with some movements or intermittent steps according to ActiPASS/Acti4 algorithm *(Skotte et al., 2014)* outside periods flagged as sleep-interval(s)	Walking slow (at a pace <100 steps/min)<br><br>Ambulatory movement (e.g. putting away dishes, moving in a small space)	Walk_Slow + Move	Ingebrigtsen et al<br>https://doi.org/10.4172/2165-7556.1000119<br><br>Skotte et al.<br>https://doi.org/10.1123/jpah.2011-0347<br><br>Tudor-Locke et al.<br>https://doi.org/10.1136/bjsports-2017-097628
Fast walking	"Fast walking" is defined as time of walking with a stepping cadence *(Skotte et al., 2014)* equal or above 100 steps/min *(Tudor-Locke et al., 2018)* according to ActiPASS/Acti4 *(Skotte et al., 2014),* outside periods flagged as sleep-interval(s)	Walking faster	Walk_Fast	
Exercise-like activities[a]	"Exercise-like activities" is defined as the time of cycling, stair-walking and running according ActiPASS/Acti4 algorithm *(Skotte et al., 2021).*	Running, cycling (can be using exercise equipment like cross-trainers or other gym equipment with periodic activity), stair-walking (can be situations where thighs are lifted higher than when walking on a flat surface, such as navigating hills or rough terrain)	Run + Stair + Cycle	Skotte et al.<br>https://doi.org/10.1123/jpah.2011-0347

Sleep + Sedentary Behavior + Standing + Slow Walking + Fast Walking + Exercise Like Activities + Other[b] + Non Wear = 24 hours<br>Full ActiPASS variable definitions can be found at : https://github.com/Ergo-Tools/ActiPASS/wiki<br>[a] Behavior **type** and not intensity was captured for this combined variable; whilst these exercise behaviors are often done at moderate-vigorous intensity, incidental activity done at varying intensities across the day is also captured (i.e. running for the bus).<br>[b]Note that some movement could not be detected and therefore is included in other (e.g. rowing, etc.). This was not included in the composition

The below table provides further details on the individual algorithms, key references and corresponding implementation source files.

Algorithm	Reference	Corresponding Implementation Source files
Automatic device calibration	Vincent van Hees et al.<br>https://doi.org/10.1152/japplphysiol.00421.2014	https://github.com/Ergo-Tools/ActiPASS/blob/main/utils/AutoCalibrate.m
Non-wear		https://github.com/Ergo-Tools/ActiPASS/blob/main/library/NotWorn.m
Individual calibration		https://github.com/Ergo-Tools/ActiPASS/blob/main/algorithms/EstimateRefThigh1.m
Detection of physical activity types	Skotte et al.<br>https://doi.org/10.1123/jpah.2011-0347	https://github.com/Ergo-Tools/ActiPASS/blob/main/library/FindAnglesAndVM.m<br>https://github.com/Ergo-Tools/ActiPASS/blob/main/algorithms/ActivityDetect.m
Stepping cadence	Ingebrigtsen et al<br>https://doi.org/10.4172/2165-7556.1000119	https://github.com/Ergo-Tools/ActiPASS/blob/main/library/findCadenceN.m
Differentiation of lying from sitting	Hettiarachchi et al.<br>https://doi.org/10.3390/s21030904	https://github.com/Ergo-Tools/ActiPASS/blob/main/algorithms/lyingAlgA.m<br>https://github.com/Ergo-Tools/ActiPASS/blob/main/algorithms/lyingAlgB.m
Times-in-bed & Sleep algorithm	Johansson et al.<br>https://doi.org/10.1111/jsr.13725	https://github.com/Ergo-Tools/ActiPASS/blob/main/algorithms/calcBedLgc.m<br>https://github.com/Ergo-Tools/ActiPASS/blob/main/algorithms/SkottesSlp.m

The below table outlines the thresholds for each brand of accelerometer used across the six studies.

**Device**	**Range**	**Resolution**	**Support sample frequency > 25Hz**	**Sensitivity (milligravity / least significant bit (LSB))**	**Validity with Acti4**
Actigraph GTX3X+	±6g	12 bit	Yes	3 mg/LSB	Yes
Axivity AX3	Selectable ±2g to ±16g	13 bit	Yes	2mg/LSB (at ±8g)	Yes
ActivPAL4 (ActivPAL Micro 4)	±4g	10 bit	Yes	8mg/LSB	Yes

There was insufficient space for the key references for software packages used in analysis. They include:

Gerald van den Boogaart K, Tolosana-Delgado R. “compositions”: A unified R package to analyze compositional data. Computers & Geosciences. 2008;34:320-338.

Hettiarachchi, P., & Johansson, P. (2024). ActiPASS (Version 1.56) [Computer software]. https://doi.org/10.5281/zenodo.7701098

Palarea-Albaladejo JM-F, J.A. zCompositions — R package for multivariate imputation of left-censored data under a compositional approach. Chemometrics and Intelligent Laboratory Systems. 2015;143:85-96.

Templ MH, K Filzmoser , P. robCompositions: An R-package for Robust Statistical Analysis of Compositional Data. In: Pawlowsky-Glahn V, Buccianti A, eds. Compositional Data Analysis: Theory and Applications. 2011.

Viechtbauer W (2010). “Conducting meta-analyses in R with the metafor package.” Journal of Statistical Software, 36(3), 1–48

Wickham H, Averick M, Bryan J, Chang W, McGowan L, François R, Grolemund G, Hayes A, Henry L, Hester J, et al. Welcome to the tidyverse. Journal of Open Source Software. 2019;4:1686.

Supplemental M2. Details of individual cohort funding

The Maastricht Study was supported by the European Regional Development Fund via OP-Zuid, the Province of Limburg, the Dutch Ministry of Economic Affairs (grant 31O.041), Stichting De Weijerhorst (Maastricht, the Netherlands), the Pearl String Initiative Diabetes (Amsterdam, the Netherlands), the Cardiovascular Center (CVC, Maastricht, the Netherlands), CARIM School for Cardiovascular Diseases (Maastricht, the Netherlands), CAPHRI Care and Public Health Research Institute (Maastricht, the Netherlands), NUTRIM School for Nutrition and Translational Research in Metabolism (Maastricht, the Netherlands), Stichting Annadal (Maastricht, the Netherlands), Health Foundation Limburg (Maastricht, the Netherlands), and by unrestricted grants from Janssen-Cilag BV (Tilburg, the Netherlands), Novo Nordisk Farma BV (Alphen aan den Rijn, the Netherlands), and Sanofi-Aventis Netherlands BV (Gouda, the Netherlands).

The 1970 British Cohort Study is funded by the Economic and Social Research Council (ES/M001660/1). The age 46 sweep was also funded by the British Heart Foundation (grant SP/15/6/31397) and a joint award from the Economic and Social Research Council and the Medical Research Council (grant RES-579-47-0001). The ALSWH is funded by the Australian Government Department of Health and Aged Care and its substudy (Menarche-to-PreMenopause), from which accelerometry and clinical data were obtained, was funded by the National Health and Medical Research Council Project Grant (APP1129592). DPhacto is funded by The Danish Working Environment Research Fund. NES is supported by the Department of Physiology of the Radboud University Medical Center, the Dutch Heart Foundation (2020T063), and Siemens Healthcare Diagnostics (the Hague, Netherlands). FIREA is supported by the Academy of Finland (286294, 294154, 319246, 332030), Ministry of Education and Culture, Juho Vainio Foundation and Finnish State Grants for Clinical Research.

Table S1. Overview of individual cohort details

Cohort	Sample description and age range	Sample size with valid accel data	Sex	Device (years; accelerometer timeline)	Leading institution, Country	Eligibility criteria	Ethics approval (committee name, reference number)
Australian Longitudinal Study on Women's Health (ALSWH)	General population / 41-49 years	n=941 with accelerometer data	Females only	ActivPAL3 and ActivPAL4 micro (2019-2021; device fitted/ given to participants at main data collection visit)	The University of Queensland and The University of Sydney, Australia	Women in the 1973-78 cohort, not pregnant, not currently undergoing treatment for breast or reproductive cancer	Metro South Health and Health Services Human Research Ethics Committee (reference number: HREC/2019/ QMS/52052)
1970 British Cohort Study (BCS70)	General population / 46 years	n=5229 with accelerometer data	Females & males	ActivPAL3 micro (2016-18; device fitted/ given to participants at main data collection visit)	University College London, United Kingdom	Born within 1 week in April 1970 in England, Scotland or Wales	NRES Committee South East Coast - Brighton and Sussex (Ref 15/LO/1446)
Danish PHysical ACTivity cohort with Objective measurements cohort (DPhacto)	Workers in cleaning, manufacturing, and transportation companies / 18-65 years	n=771 with accelerometer data	Females & males	Actigraph GT3X (2012-13; device fitted/ given to participants at main data collection visit)	National Research Centre for the Working Environment, Denmark	Workers from manual-based jobs in manufacturing, transportation and cleaning sectors	Danish data protection agency and local Ethics Committee (H-2-2012-011).

Finnish Retirement and Aging Study (FIREA)	General Population, public sector employees / 59-65 years	n=253 with accelerometer data	Females & males	Axivity (2015-18; device fitted/ given to participants at main data collection visit)	University of Turku, Finland	Public sector employees whose statutory retirement date was between 2014 and 2019	Ethics Committee of Hospital District of Southwest Finland.
Nijmegen Exercise Study (NES)	Participants in Nijmegen 4-day Marches or Seven Hills Run, and their friends & family members / 23-87+ years	n=537 with accelerometer data	Females & males	ActivPAL3 micro (2021; device fitted/ given to participants at main data collection visit)[b]	Radboud university medical centre, Netherlands	Individuals participating in Dutch sport events (i.e. International Nijmegen Four Days Marches and the Seven Hills Run) and their family and friends.	The Local Ethics Committee on Research Involving Human Subjects (CMO) of the region Arnhem and Nijmegen, the Netherlands (NL36743.091.11)
The Maastricht Study (TMS)	General Population (Oversampling of those with T2 Diabetes) / 40-75 years	n=7515 with accelerometer data	Females& males	ActivPAL3 (2010-17; device fitted/ given to participants at main data collection visit)	Maastricht University Medical Center+ and Maastricht University, Netherlands	Individuals aged 40-75 years old, oversampling of those with Type 2 Diabetes	Institutional medical ethical committee (NL31329.068.10) and the Minister of Health, Welfare and Sports of the Netherlands (Permit 131088-105234-PG)

[a] Some participants were sent the accelerometer via mail and provided with fitting instructions.

[b] Although participants were given the accelerometer device at the main clinic visit, a small amount were given the device up to 1.5 weeks before (due to personal conflicts such as holiday, scheduled medical events, etc) or up to 1 month after (due to limited activPAL availability or 2nd wear due to collection/processing error)

Table S2. Assessment and harmonisation of blood pressure and covariate measures by cohort

Variable name	***Variable description and coding***	***AWH***	***BCS70***	***DPHACTO***	***FIREA***	***NES***	***The Maastricht Study***
**OUTCOMES**							
SBP and DBP	Continuous measure (mmHg)	Mean of 2nd and 3rd measurements taken after a 5-min seated rest period using an automated blood pressure monitor (arm not recorded in protocol, although same arm used for all measurements)<br><br>**Models:**[a]<br>QLD: Vital Signs Machine 6000 Series<br>VIC: Welch Allyn Connex® ProBP™ 3400<br>SA: Phillips MP30<br>WA: Omron HEM-907<br>NSW: Omron HEM-7121	Mean of 3 measurements on the right arm after a 5-min seated rest period using an Omron HEM 907 blood pressure monitor at 1-minutes intervals.	Mean of 3 measurements on the right arm after a 10-minute seated rest period, using an Omron M6 Comfort monitor.	Mean of 2 measurements (1 left, 1 right) after a 5-minute seated rest period, using the Microlife Watch BP Office Central monitor.	Mean of 3 measurements (2 left, 1 right) after a 10-min lying rest period using Omron M3 monitor<br><br>If differences between measures were >10 mmHg for SBP or >5 mmHg for DBP, a 4th measurement was taken on the right arm and all 4 measurements were used to calculate average.	Mean of 3 measurements on the right arm after a 10-minute seated rest period, using an Omron 705IT monitor.<br><br>If differences between 2nd and 3rd measure >10mmHg, a 4th measurement was performed and all 4 measurements were used to calculate average.
COVARIATES							
Age	Continuous (years)	Question: "What is your age in years?"	All participants assigned age 46 (birth cohort study; year of birth: 1970; year of accelerometer assessment: 2016)	Determined using workers' unique civil registration number based on time between date of birth and date of measurement visit	Derived based on time between date of birth and date of measurement visit	Derived based on time between date of birth and date of measurement visit	Derived based on time between date of birth and date of measurement visit

Variable name	*Variable description and coding*	*AWH*	*BCS70*	*DPHACTO*	*FIREA*	*NES*	*The Maastricht Study*
Sex	1: Male<br>2: Female	2: Female	1: Male<br>2: Female	1: Male<br>2: Female	1: Male<br>2: Female	1: Male<br>2: Female	1: Male<br>2: Female
Smoking	0: Non-smoker<br>1: Smoker	**Question**: "How often do you currently smoke?"<br>**Responses & coding**:<br>0: Not at all<br>1: Daily; At least weekly (but not daily); Less often than weekly	**Question**: "Which of the statements on this card applies to you?"<br>**Responses & coding**:<br>0: I've never smoked cigarettes; I used to smoke but don't at all<br>1: I now smoke occasionally but not daily; I smoke cigarettes every day	**Question**: "Do you smoke?"<br>**Responses & coding**:<br>0: Never smoked; Formerly smoked<br>1: Daily smoking; Occasionally smoking	**Question**: "Do you currently smoke or have you smoked regularly, i.e. daily or almost daily?"<br>**Responses & coding**:<br>0: No I have never smoked; Yes, previously<br>1: Yes, currently	**Question**: "Do you smoke?"<br>**Responses & coding**:<br>0: No, but I smoked in the past; No, I have never smoked<br>1: Yes	**Question**: "Do you smoke?"<br>**Responses & coding**:<br>0: No I have never smoked; No, I stopped smoking more than 6 months ago; No, I stopped less than 6 months ago<br>1: Yes
Alcohol consumption	1: Lowest tertile<br>2: Middle tertile<br>3: Highest tertile<br><br>(cohort-specific tertiles)	**Question**: "How often do you normally drink alcohol?"<br>**Responses & coding**:<br>1: Less than once a week<br>2: 1-4 days/week<br>3: 5+ days/week	Units consumed in last 7 days (continuous)<br><br>Derived variable from interview questions on type, size and number of alcoholic drinks	**Question**: "Do you drink alcohol? How many units did you drink last week?"<br><br>(continuous)	Total intake in grams/week (continuous)<br><br>Derived from questionnaire items on amount of beer, wine and spirits	**Question**: "How many glasses did you consumed on average per week in the past year?"<br><br>(continuous)	Total intake in grams/day (continuous)<br><br>Derived from Food Frequency Questionnaire with a 1-year reference period
COVARIATES (incl in sensitivity only)							
Body mass index	Continuous measure (kg/m^2)	Derived from clinical measurement of height (stadiometer) and weight (digital scale)<br><br>Stadiometer models:[a]<br>QLD: ADE (no name)	Derived from clinical measurement of height (without shoes; portable Leicester stadiometer and weight (Tanita BF-522W scales)	Derived from clinical measurement of height (without shoes; stadiometer- Seca, model 213) and weight (Tanita bio-impedance segmental body composition	Derived from clinical measurement of height (without shoes) and weight using Inbody 720 scale (Biospace Co., Seoul, Korea)	Derived from clinical measurement of height (without shoes) and weight (Seca 881)	Derived from clinical measurement of height (stadiometer – Seca 222) and weight (Seca 877)

Variable name	*Variable description and coding*	*AWH*	*BCS70*	*DPHACTO*	*FIREA*	*NES*	*The Maastricht Study*
		VIC: Seca BE35208<br>SA: Seca (no name)<br>WA: ADE MZ10023<br>NSW: ADE (no name)<br><br>Weigh scale models:[a]<br>QLD: Seca 813<br>VIC: Seca BE38844<br>SA: Soehnle EB9373<br>WA: Perma Lifestyle Professional Slimline Body Monitor<br>NSW: Seca 813		analyzer- model BC418 MA)			
Education	**0**: None or lower than high school<br>**1**: High school qualifications (age 16y)<br>**2**: Further education qualifications (age 16-18y)<br>**3**: university degree and higher (18+y)	**Question**: "What is the highest level of qualification you have completed?"<br><br>**Responses & coding**:<br>**0**: No formal qualifications<br>**1**: Year 10 or equivalent<br>**2**: Year 12 or equivalent, Trade/apprenticeship, Certificate/Diploma<br>**3**: University degree, Higher university degree	**Derived variable** of National Vocational Qualifications categories based on self-reported "recognised academic, vocational, clerical, business or commercial qualifications" asked at each wave<br><br>**Responses & coding**:<br>**0:** No academic qualification<br>**1:** GCDS D-E, GCSE A-C, CSES 2-5, Other Scottish qualifications, Good O levels Scottish	N/A	N/a	Question: "Please select your highest educational qualification from the list below"<br><br>Responses & coding:<br>0: lower education (primary school), lower pre-vocational education (low)<br>1: pre-vocational education (moderate), secondary education (moderate)<br>2: middle-level applied education (moderate)	**Question**: "What is your highest completed educational level?"<br><br>**Responses & coding**:<br>0: None, Uncompleted primary educational level, Primary educational level<br>1: Lower vocational education, Intermediate general secondary education<br>2: Intermediate vocational education, Higher general secondary education,

Variable name	*Variable description and coding*	*AWH*	*BCS70*	*DPHACTO*	*FIREA*	*NES*	*The Maastricht Study*
			standards<br>**2**: As levels or 1 A level; 2+ A levels, Scottish higher/6th, diploma<br>**3**: Degree level, Higher degree			3: higher professional education (high), university (high)	Higher vocational education<br>3: University education
<u>*Occupational class*</u>	0: Not working<br>1: Low occupational class<br>2: Intermediate occupational class<br>3: High occupational class	**Question**: "What is your main occupation now?"<br><br>**Responses & coding**:<br><br>0: No paid job<br>1: Elementary clerical, sales or service worker; Labourer or related worker<br>2: Tradesperson or related worker; Advanced clerical or service worker; Intermediate clerical; sales/service worker; Intermediate production or transport worker<br>3: Manager or administrator; Professional;	**Derived variable** of National Statistics Socio-economic Classification (NS-SEC) based on participants "[description] in [their] own words what [they] mainly did in this job]<br><br>**Responses & coding**:<br>0: Never worked and long-term unemployed<br>1: L10 Lower supervisory occupations; L11 Lower technical occupations; L12 Semi-routine occupations; L13 Routine occupations	**Derived variable** pulled from personnel lists of companies:<br><br>**Coding:**<br>1= production worker AND unskilled<br>2=production worker AND skilled<br>2= Administration and office workers AND skilled or unskilled	**Derived variable** from the Register of Pension Institute Keva of International Standard Classification of Occupations (ISCO)<br><br>**Responses & coding**:<br>1: plant and machine operators, and assemblers; elementary occupations<br>2: clerical support workers; service and sales workers; skilled agricultural, forestry and fishery workers; craft and related trades workers<br>3: managers; professionals; technicians and	N/a	**Question**: "Which category best fits your current/past job?"<br><br>**Responses & coding**:<br>0: Not working<br>1: Low occupational class<br>2: Intermediate occupational class; Self-employed<br>3: High occupational class, Professionals

Variable name	*Variable description and coding*	*AWH*	*BCS70*	*DPHACTO*	*FIREA*	*NES*	*The Maastricht Study*
		Associate professional	2: L5 Lower managerial and administrative; L6 Higher supervisory occupations; L7 Intermediate occupations; L8 Employers in small organisations; L9 Own account workers<br>3: L1 Employers in large establishments; L2 Higher managerial and administrative; L3 Higher professional occupations; L4 Lower professional and higher technical		associate professionals		
<u>*Mobility limitations*</u>	Continuous score from 0 to 100 of the SF 10-item physical function, where 0 indicates poor mobility and 100 indicates no mobility problems.	Available in ALSWH, BCS70, FIREA and TMS only.<br>Each used the SF-36 scale. The 10-items included limitations in: vigorous activities, moderate activities, lifting and carrying groceries, climbing several flights of stairs, climbing one flight of stairs, bending, kneeling or stooping, walking about two kilometers, walking about a half kilometer, in walking about 100 metres, in bathing or dressing. Each item had three possible responses: Yes, limited a lot (0); Yes, limited a little (50); No, not limited at all (100). Mobility limitations score was calculated as the average score across all ten items.					
OTHER RELEVANT VARIABLES							
<u>*History of cardiovascular conditions*</u>	0: No history of CVD<br>1: History of CVD	**Questions**:<br>Wave 1:"Have you ever been told by a doctor that you have heart disease"	**Questions**: "Since last collection wave, have you had any of the health problems listed on this card?	**Question**: "Do you have angina pectoris?"	**Questions**: "Has a doctor/physician given you a diagnosis of [angina	**Questions**: "Which type of diseases below has been diagnosed by physician?	**Questions**: Rose Questionnaire<br><br>Responses:

Variable name	*Variable description and coding*	*AWH*	*BCS70*	*DPHACTO*	*FIREA*	*NES*	*The Maastricht Study*
		Wave 2: "[In the last 4 years] [In more than 4 years ago], have you ever been told by a doctor that you have heart disease?" Waves 3-8: "In the last 3 years have you been diagnosed or treated for heart disease?" **Responses & coding**: 0: No to all of above 1: Yes to any of above	[high blood pressure]" "Since last collection wave, have you had any of the health problems listed on this card? Please include any health problems that had already started before that date. [heart problems]/ [stroke]". **Responses & coding**: 0: No to all of above 1: Yes to any of above	**Responses & coding**: 0: No 1: Yes	pectoris]/[myocardial infarction]/[stroke]?" **Responses & coding**: 0: No to all of above 1: Yes to any of above	[myocardial infarction]/ [heart failure]/ [stroke]/ [atrial fibrillation]?" **Responses & coding**: 0: No to all of above 1: Yes to any of above	1: Selected any of myocardial infarction - cerebrovascular infarction and/or hemorrhage - percutaneous artery angioplasty of the coronary arteries, abdominal arteries, peripheral arteries or carotid artery - vascular surgery on coronary arteries, abdominal arteries, peripheral arteries or carotid artery. 0: selected none of the above
Medication use	0: No anti-hypertensive medications 1: Any anti-hypertensive medications	**Protocol:** Participants were asked to bring all medications to the assessment, which were coded using Anatomical Therapeutic Chemical classification 1: Any of C02 anti-hypertensive drug	**Protocol:** Research nurses collected data on all prescription medications which were coded using British National Formulary edition 69 codes **Responses & coding**: 1: Any of 0201 – 0207: hypertension related drugs	**Questions**: "Have you in the last three months been taken prescription medication?" "If yes, what kind of medication?" **Responses & coding**: 1: Antihypertensive 0: No medications or other medications	**Protocol:** Research nurses inquired about all prescription medications, which were coded using Anatomical Therapeutic Chemical classification 1: Any of C02 anti-hypertensive drug	**Questions**: "Did you use medication in the past year?" was asked immediately following positive responses to "Which of the following diseases below has been diagnosed by physician?" for 2) hypertension; **Responses & coding**: 1: Yes for any of above	**Protocol:** Participants were asked to bring all medications to the assessment, which were coded using Anatomical Therapeutic Chemical classification 1: Any of C02 anti-hypertensive drug

Variable name	*Variable description and coding*	*AWH*	*BCS70*	*DPHACTO*	*FIREA*	*NES*	*The Maastricht Study*
				**Questions:** "Do you take medication for high blood pressure?<br>**Responses & coding**:<br>1: Yes<br>2: No		0: No	

QLD: Queensland VIC: Victoria SA: South Australia WA: West Australia NSW: New South Wales

Table S3. Cohort-stratified means for 24-hour movement behaviors and BP outcomes

	TMS (n=759)	*ALSWH (n=425)*	*BCS (n=5212)*	**DPhacto (n=830)**	**FIREA (n=248)**	**NES (n=537)**
**Mean ±SD**						
**24- hour movement behaviors**						
Sleep	424 ±107	447 ±99.1	439 ±114	395 ±129	409 ±115	413 ±96.5
Sedentary behavior	656 ±69.7	624 ±60.4	633 ±73.2	589 ±73.2	627 ±56.7	655 ±66.3
Stand	184 ±60.6	201 ±61.8	194 ±69.5	231 ±75.9	214 ±70.8	161 ±55.8
Slow walk	92.1 ±30.3	84 ±27.1	92.4 ±35.6	126 ±49.6	103 ±36	91.9 ±28.2
Fast walk	65 ±25.5	74.6 ±24.3	67.1 ±26.5	82.5 ±30.9	68.5 ±22.7	77.4 ±35.2
Combined exercise	17.9 ±17.1	9.59 ±8.81	11.7 ±11.1	14.2 ±13.7	13.1 ±10.9	39.9 ±28.4
**Systolic blood pressure (mmHg)***	137 ±20	119 ±12.5	125 ±15.5	135 ±15.7	138 ±17.3	140 ±19.1
**Diastolic blood pressure (mmHg)***	79 ±11.6	77.6 ±8.88	77.6 ±11.5	85.2 ±11.3	84 ±9.4	84.3 ±10.6

Table S4. Estimated regression coefficients [a] of isometric log ratios of each behavior (ILR coordinate representing 1 behavior relative to other 6) and **systolic blood pressure**

	Maximal sample	Complete cases sample		Maximal adjustment sample [b]		
**BEHAVIOR**	Model 1: age, sex, cohort adjusted	Model 1: age, sex, cohort adjusted	Model 2: age, sex, cohort, smoking, alcohol adjusted	Model 1: age, sex, cohort adjusted	Model 2: age, sex, cohort, smoking, alcohol adjusted	Model 3: age, sex, cohort, smoking, alcohol, education, employment, mobility adjusted
Sample size	n=14761	n=12651		n=9799		
**Sleep**	-2.95 (-4.12, -1.77)	-2.43 (-3.73, -1.13)	-2.47 (-3.77, -1.16)	-2.34 (-3.84, -0.85)	-2.36 (-3.86, -0.86)	-2.41 (-3.90, -0.92)
**Sedentary behavior**	4.58 (3.55, 5.61)	4.18 (3.04, 5.31)	4.31 (3.18, 5.45)	3.79 (2.48, 5.10)[ns]	3.90 (2.59, 5.21)	4.10 (2.77, 5.42)
**Standing**	0.35 (-0.73, 1.43)[ns]	0.31 (-0.87, 1.49)[ns]	0.29 (-0.89, 1.47)[ns]	-0.10 (-1.46, 1.27)[ns]	-0.06 (-1.43, 1.31)[ns]	-0.03 (-1.39, 1.33)[ns]
**Slow walking**	-0.50 (-1.66, 0.65)[ns]	-0.54 (-1.80, 0.71)[ns]	-0.34 (-1.59, 0.92)[ns]	0.04 (-1.40, 1.49)[ns]	0.19 (-1.26, 1.64)[ns]	-0.83 (-2.29, 0.63)[ns]
**Fast walking**	0.45 (-0.40, 1.30)[ns]	0.30 (-0.62, 1.23)[ns]	0.13 (-0.80, 1.06)[ns]	0.54 (-0.53, 1.61)[ns]	0.37 (-0.71, 1.44)[ns]	0.66 (-0.41, 1.74)[ns]
**Combined exercise-like**	-1.93 (-2.28, -1.57)	-1.81 (-2.19, -1.43)	-1.93 (-2.32, -1.55)	-1.94 (-2.37, -1.51)	-2.03 (-2.47, -1.59)	-1.49 (-1.94, -1.04)

[a] Linear regression. Coefficients indicate change in blood pressure per 1 unit increase in the corresponding ILR coordinate. Value >0 indicates more time spent in the behavior relative to others is associated with higher blood pressure; value <0 indicates that more time spent in the behavior relative to others is associated with lower blood pressure. Coefficients indicate the presence of an association, but effect size is not directly interpretable due to the isometric log-ratio transformation.

[b] Restricted to three cohorts: ALSWH, BCS70, TMS

[ns] indicates non-significant results; all other estimates are significant at $p<0.05$

Table S5. Estimated regression coefficients[a] of isometric log ratios of each behavior (ILR coordinate representing 1 behavior relative to other 6) and **diastolic blood pressure**

BEHAVIOR	Maximal sample	Complete cases sample		Maximal adjustment sample [b]		
	Model 1: age, sex, cohort adjusted	Model 1: age, sex, cohort adjusted	Model 2: age, sex, cohort, smoking, alcohol adjusted	Model 1: age, sex, cohort adjusted	Model 2: age, sex, cohort, smoking, alcohol adjusted	Model 3: age, sex, cohort, smoking, alcohol, education, employment, mobility adjusted)
Sample size	n=14761	n=12651		n=9799		
**Sleep**	-1.50 (-2.28, -0.73)	-1.32 (-2.17, -0.46)	-1.33 (-2.18, -0.48)	-1.25 (-2.24, -0.27)	-1.25 (-2.23, -0.27)	-1.24 (-2.22, -0.26)
**Sedentary behavior**	4.17 (3.49, 4.85)	3.87 (3.13, 4.61)	3.97 (3.22, 4.71)	4.00 (3.14, 4.86)	4.08 (3.22, 4.94)	4.14 (3.27, 5.01)
**Standing**	-1.51 (-2.23, -0.80)	-1.42 (-2.19, -0.65)	-1.43 (-2.21, -0.66)	-1.63 (-2.53, -0.74)	-1.61 (-2.51, -0.72)	-1.61 (-2.50, -0.72)
**Slow walking**	-0.58 (-1.35, 0.18)[ns]	-0.52 (-1.35, 0.30)[ns]	-0.38 (-1.20, 0.45)[ns]	-0.36 (-1.31, 0.59)[ns]	-0.25 (-1.20, 0.70)[ns]	-0.95 (-1.91, 0.00)[ns]
**Fast walking**	0.82 (0.25, 1.38)	0.68 (0.07, 1.28)	0.55 (-0.06, 1.16)[ns]	0.67 (-0.03, 1.37)[ns]	0.52 (-0.18, 1.23)[ns]	0.75 (0.04, 1.45)
**Combined exercise-like**	-1.39 (-1.62, -1.16)	-1.29 (-1.53, -1.04)	-1.37 (-1.63, -1.12)	-1.42 (-1.70, -1.14)	-1.49 (-1.78, -1.21)	-1.09 (-1.38, -0.79)

[a] Linear regression. Coefficients indicate change in blood pressure per 1 unit increase in the corresponding ILR coordinate. Value >0 indicates more time spent in the behavior relative to others is associated with higher blood pressure; value <0 indicates that more time spent in the behavior relative to others is associated with lower blood pressure. Coefficients indicate the presence of an association, but effect size is not directly interpretable due to the isometric log-ratio transformation.
[b] Restricted to three cohorts: ALSWH, BCS70, TMS
[ns] indicates non-significant results; all other estimates are significant at $p<0.05$

Table S6. Estimated regression coefficients[a] of isometric log ratios of each behavior (ILR coordinate representing one behavior relative to other five) and **systolic blood pressure** with **5mmHg and 15mmHg transformation** for those currently on anti-hypertensive medication (per Tobin et al.,)

	Maximal sample	Complete cases sample		Maximal adjustment sample [b]		
**BEHAVIOR**	Model 1: age, sex, cohort adjusted	Model 1: age, sex, cohort adjusted	Model 2: age, sex, cohort, smoking, alcohol adjusted	Model 1: age, sex, cohort adjusted	Model 2: age, sex, cohort, smoking, alcohol adjusted	Model 3: age, sex, cohort, smoking, alcohol, education, employment, mobility adjusted)
Sample size	n=14761	n=12651		n=9799		
**SYSTOLIC BLOOD PRESSURE** (+5mmHg for those on anti-hypertensive medications)						
**Sleep**	-2.69 (-3.82, -1.55)	-2.26 (-3.51, -1.01)	-2.28 (-3.53, -1.03)	-2.06 (-3.50, -0.63)	-2.07 (-3.51, -0.64)	-2.11 (-3.54, -0.67)
**Sedentary behavior**	3.90 (2.91, 4.89)	3.56 (2.47, 4.65)	3.70 (2.61, 4.79)	3.05 (1.79, 4.31)	3.17 (1.91, 4.42)	3.41 (2.14, 4.69)
**Standing**	0.44 (-0.60, 1.47) [ns]	0.38 (-0.76, 1.52) [ns]	0.36 (-0.77, 1.50) [ns]	-0.05 (-1.37, 1.26) [ns]	-0.02 (-1.33, 1.30) [ns]	0.00 (-1.31, 1.31) [ns]
**Slow walking**	-0.73 (-1.84, 0.38) [ns]	-0.73 (-1.93, 0.48) [ns]	-0.51 (-1.72, 0.69) [ns]	-0.13 (-1.52, 1.26) [ns]	0.02 (-1.37, 1.41) [ns]	-0.87 (-2.28, 0.53) [ns]
**Fast walking**	0.70 (-0.12, 1.52) [ns]	0.56 (-0.33, 1.45) [ns]	0.37 (-0.52, 1.26) [ns]	0.82 (-0.21, 1.85) [ns]	0.63 (-0.40, 1.67) [ns]	0.84 (-0.19, 1.88) [ns]
**Combined exercise-like**	-1.62 (-1.96, -1.29)	-1.51 (-1.88, -1.15)	-1.64 (-2.01, -1.27)	-1.63 (-2.05, -1.22)	-1.72 (-2.14, -1.30)	-1.27 (-1.70, -0.84)
**SYSTOLIC BLOOD PRESSURE** (+15mmHg for those on anti-hypertensive medications)						
**Sleep**	-3.21 (-4.44, -1.97)	-2.60 (-3.97, -1.24)	-2.65 (-4.01, -1.28)	-2.62 (-4.20, -1.05)	-2.65 (-4.22, -1.08)	-2.71 (-4.28, -1.14)
**Sedentary behavior**	5.25 (4.17, 6.33)	4.79 (3.60, 5.98)	4.93 (3.74, 6.12)	4.53 (3.16, 5.91)	4.64 (3.26, 6.02)	4.78 (3.39, 6.18)
**Standing**	0.26 (-0.87, 1.39) [ns]	0.23 (-1.01, 1.47) [ns]	0.22 (-1.02, 1.46) [ns]	-0.14 (-1.58, 1.30) [ns]	-0.11 (-1.54, 1.33) [ns]	-0.07 (-1.50, 1.36) [ns]
**Slow walking**	-0.27 (-1.48, 0.94) [ns]	-0.36 (-1.68, 0.95) [ns]	-0.16 (-1.48, 1.16) [ns]	0.22 (-1.30, 1.74) [ns]	0.35 (-1.17, 1.88) [ns]	-0.78 (-2.32, 0.75) [ns]
**Fast walking**	0.19 (-0.70, 1.08) [ns]	0.05 (-0.92, 1.02) [ns]	-0.11 (-1.09, 0.86) [ns]	0.26 (-0.87, 1.39) [ns]	0.10 (-1.03, 1.23) [ns]	0.49 (-0.65, 1.62) [ns]
**Combined exercise-like**	-2.23 (-2.60, -1.86)	-2.11 (-2.51, -1.71)	-2.23 (-2.63, -1.83)	-2.25 (-2.70, -1.79)	-2.33 (-2.79, -1.87)	-1.71 (-2.18, -1.24)

[a] Linear regression. Coefficients indicate change in blood pressure per 1 unit increase in the corresponding ILR coordinate. Value >0 indicates more time spent in the behavior relative to others is associated with higher blood pressure; value <0 indicates that more time spent in the behavior relative to others is associated with lower blood pressure. Coefficients indicate the presence of an association, but effect size is not directly interpretable due to the isometric log-ratio transformation.

[b] Restricted to three cohorts: ALSWH, BCS70, TMS

[ns] indicates non-significant results; all other estimates are significant at $p<0.05$

Table S7. Estimated regression coefficients[a] of isometric log ratios of each behavior (ILR coordinate representing 1 behavior relative to other 6) and **systolic blood pressure** in subsamples of those **not on anti-hypertensive medications** and those **without history of cardiovascular disease**

BEHAVIOR	Maximal sample	Complete cases sample		Maximal adjustment sample [b]		
	Model 1: age, sex, cohort adjusted	Model 1: age, sex, cohort adjusted	Model 2: age, sex, cohort, smoking, alcohol adjusted	Model 1: age, sex, cohort adjusted	Model 2: age, sex, cohort, smoking, alcohol adjusted	Model 3: age, sex, cohort, smoking, alcohol, education, employment, mobility adjusted)
**SYSTOLIC BLOOD PRESSURE (not on anti-hypertensive medications)**						
Sample size	n=11394	n=9563		n=7365		
**Sleep**	-2.28 (-3.51, -1.05)	-2.43 (-3.80, -1.05)	-2.44 (-3.81, -1.06)	-2.25 (-3.84, -0.66)	-2.21 (-3.80, -0.62)	-2.28 (-3.87, -0.70)
**Sedentary behavior**	2.90 (1.84, 3.96)	2.92 (1.75, 4.09)	3.02 (1.85, 4.19)	2.35 (0.99, 3.71)	2.40 (1.04, 3.76)	2.80 (1.42, 4.17)
**Standing**	0.68 (-0.40, 1.76)[ns]	0.90 (-0.29, 2.09)[ns]	0.88 (-0.31, 2.07)[ns]	0.66 (-0.72, 2.03)[ns]	0.67 (-0.71, 2.04)[ns]	0.68 (-0.69, 2.05)[ns]
**Slow walking**	-1.03 (-2.19, 0.14)[ns]	-0.93 (-2.20, 0.34)[ns]	-0.70 (-1.97, 0.57)[ns]	-0.34 (-1.81, 1.12)[ns]	-0.21 (-1.68, 1.26)[ns]	-0.87 (-2.35, 0.61)[ns]
**Fast walking**	0.88 (0.00, 1.75)[ns]	0.60 (-0.36, 1.56)[ns]	0.43 (-0.53, 1.39)[ns]	0.84 (-0.27, 1.96)[ns]	0.67 (-0.44, 1.79)[ns]	0.68 (-0.43, 1.80)[ns]
**Combined exercise-like**	-1.15 (-1.52, -0.79)	-1.07 (-1.47, -0.67)	-1.19 (-1.59, -0.79)	-1.25 (-1.70, -0.80)	-1.32 (-1.78, -0.87)	-1.01 (-1.47, -0.54)
**SYSTOLIC BLOOD PRESSURE (no history of CVD)**						
Sample size	n=13184	n=11277		n=8711		
**Sleep**	-2.99 (-4.24, -1.75)	-2.65 (-4.03, -1.26)	-2.67 (-4.06, -1.29)	-2.47 (-4.08, -0.87)	-2.46 (-4.06, -0.86)	-2.52 (-4.11, -0.92)
**Sedentary behavior**	4.65 (3.57, 5.74)	4.42 (3.23, 5.61)	4.54 (3.35, 5.73)	3.94 (2.55, 5.33)	4.02 (2.63, 5.41)	4.35 (2.95, 5.75)
**Standing**	0.50 (-0.62, 1.63)[ns]	0.50 (-0.73, 1.74)[ns]	0.47 (-0.76, 1.71)[ns]	-0.16 (-1.60, 1.28)[ns]	-0.14 (-1.58, 1.29)[ns]	-0.08 (-1.51, 1.35)[ns]
**Slow walking**	-0.60 (-1.80, 0.61)[ns]	-0.58 (-1.88, 0.73)[ns]	-0.36 (-1.67, 0.95)[ns]	0.15 (-1.36, 1.66)[ns]	0.28 (-1.24, 1.80)[ns]	-0.85 (-2.39, 0.68)[ns]
**Fast walking**	0.22 (-0.68, 1.11)[ns]	0.00 (-0.98, 0.98)[ns]	-0.17 (-1.15, 0.81)[ns]	0.38 (-0.76, 1.53)[ns]	0.22 (-0.92, 1.37)[ns]	0.47 (-0.68, 1.61)[ns]
**Combined exercise-like**	-1.78 (-2.16, -1.41)	-1.70 (-2.10, -1.30)	-1.81 (-2.22, -1.41)	-1.84 (-2.30, -1.38)	-1.92 (-2.38, -1.46)	-1.37 (-1.85, -0.90)

[a] Linear regression. Coefficients indicate change in blood pressure per 1 unit increase in the corresponding ILR coordinate. Value >0 indicates more time spent in the behavior relative to others is associated with higher blood pressure; value <0 indicates that more time spent in the behavior relative to others is associated with lower blood pressure. Coefficients indicate the presence of an association, but effect size is not directly interpretable due to the isometric log-ratio transformation.
[b] Restricted to three cohorts: ALSWH, BCS70, TMS
[ns] indicates non-significant results; all other estimates are significant at $p<0.05$

Table S8. Estimated regression coefficients[a] of isometric log ratios of each behavior (ILR coordinate representing 1 behavior relative to other 6) and **diastolic blood pressure** with **5mmHg and 15mmHg transformation** for those currently on anti-hypertensive medication (per Tobin et al., 2005)

	**Maximal sample**	**Complete cases sample**		**Maximal adjustment sample** [b]		
**BEHAVIOR**	Model 1: age, sex, cohort adjusted	Model 1: age, sex, cohort adjusted	Model 2: age, sex, cohort, smoking, alcohol adjusted	Model 1: age, sex, cohort adjusted	Model 2: age, sex, cohort, smoking, alcohol adjusted	Model 3: age, sex, cohort, smoking, alcohol, education, employment, mobility adjusted)
Sample size	n=14761	n=12651		n=9799		
**DIASTOLIC BLOOD PRESSURE** (+5mmHg for those on anti-hypertensive medications)						
**Sleep**	-1.24 (-1.97, -0.51)	-1.14 (-1.94, -0.35)	-1.15 (-1.95, -0.35)	-0.97 (-1.89, -0.06)	-0.96 (-1.87, -0.05)	-0.94 (-1.85, -0.03)
**Sedentary behavior**	3.50 (2.86, 4.14)	3.25 (2.56, 3.95)	3.35 (2.66, 4.04)	3.25 (2.45, 4.06)	3.34 (2.54, 4.14)	3.45 (2.64, 4.26)
**Standing**	-1.43 (-2.09, -0.76)	-1.35 (-2.07, -0.62)	-1.36 (-2.08, -0.64)	-1.59 (-2.43, -0.75)	-1.57 (-2.40, -0.73)	-1.58 (-2.41, -0.75)
**Slow walking**	-0.81 (-1.53, -0.09)	-0.71 (-1.48, 0.06) [ns]	-0.55 (-1.32, 0.22) [ns]	-0.53 (-1.41, 0.36) [ns]	-0.42 (-1.30, 0.47) [ns]	-1.00 (-1.89, -0.11) [ns]
**Fast walking**	1.07 (0.55, 1.60)	0.93 (0.37, 1.50)	0.79 (0.22, 1.36)	0.95 (0.29, 1.61)	0.79 (0.13, 1.44)	0.93 (0.27, 1.59)
**Combined exercise-like**	-1.09 (-1.31, -0.87)	-0.99 (-1.22, -0.75)	-1.08 (-1.31, -0.84)	-1.11 (-1.38, -0.85)	-1.19 (-1.45, -0.92)	-0.87 (-1.14, -0.59)
**DIASTOLIC BLOOD PRESSURE** (+15mmHg for those on anti-hypertensive medications)						
**Sleep**	-1.76 (-2.61, -0.92)	-1.49 (-2.42, -0.56)	-1.51 (-2.44, -0.58)	-1.53 (-2.61, -0.46)	-1.54 (-2.61, -0.46)	-1.54 (-2.61, -0.48)
**Sedentary behavior**	4.85 (4.11, 5.59)	4.49 (3.68, 5.30)	4.58 (3.77, 5.39)	4.74 (3.80, 5.68)	4.82 (3.88, 5.76)	4.83 (3.88, 5.77)
**Standing**	-1.60 (-2.38, -0.83)	-1.49 (-2.34, -0.65)	-1.50 (-2.35, -0.66)	-1.68 (-2.66, -0.70)	-1.66 (-2.64, -0.68)	-1.64 (-2.61, -0.67)
**Slow walking**	-0.35 (-1.18, 0.48) [ns]	-0.34 (-1.24, 0.55) [ns]	-0.20 (-1.10, 0.70) [ns]	-0.18 (-1.22, 0.85) [ns]	-0.08 (-1.12, 0.96) [ns]	-0.91 (-1.95, 0.13) [ns]
**Fast walking**	0.56 (-0.05, 1.17) [ns]	0.42 (-0.24, 1.08) [ns]	0.31 (-0.36, 0.97) [ns]	0.39 (-0.38, 1.16) [ns]	0.25 (-0.52, 1.03) [ns]	0.57 (-0.20, 1.34) [ns]
**Combined exercise-like**	-1.69 (-1.95, -1.44)	-1.59 (-1.86, -1.31)	-1.67 (-1.95, -1.40)	-1.73 (-2.04, -1.42)	-1.80 (-2.11, -1.48)	-1.30 (-1.62, -0.99)

[a] Linear regression. Coefficients indicate change in blood pressure per 1 unit increase in the corresponding ILR coordinate. Value >0 indicates more time spent in the behavior relative to others is associated with higher blood pressure; value <0 indicates that more time spent in the behavior relative to others is associated with lower blood pressure. Coefficients indicate the presence of an association, but effect size is not directly interpretable due to the isometric log-ratio transformation.

[b] Restricted to three cohorts: ALSWH, BCS70, TMS

[ns] indicates non-significant results; all other estimates are significant at $p<0.05$

Table S9. Estimated regression coefficients[a] of isometric log ratios of each behavior (ILR coordinate representing 1 behavior relative to other 6) and **diastolic blood pressure** in subsamples of those **not on anti-hypertensive medications** and those **without history of cardiovascular disease**

BEHAVIOR	Maximal sample	Complete cases sample		Maximal adjustment sample [b]		
	Model 1: age, sex, cohort adjusted	Model 1: age, sex, cohort adjusted	Model 2: age, sex, cohort, smoking, alcohol adjusted	Model 1: age, sex, cohort adjusted	Model 2: age, sex, cohort, smoking, alcohol adjusted	Model 3: age, sex, cohort, smoking, alcohol, education, employment, mobility adjusted)
**DIASTOLIC BLOOD PRESSURE (not on anti-hypertensive medications)**						
Sample size	n=11394	n=9563		n=7365		
**Sleep**	-0.71 (-1.54, 0.11)	-0.98 (-1.89, -0.08)	-0.99 (-1.90, -0.09)	-0.68 (-1.73, 0.37)	-0.65 (-1.69, 0.40)	-0.65 (-1.69, 0.40)
**Sedentary behavior**	2.67 (1.96, 3.38)	2.65 (1.88, 3.43)	2.72 (1.94, 3.49)	2.55 (1.65, 3.45)	2.59 (1.70, 3.49)	2.75 (1.84, 3.65)
**Standing**	-0.98 (-1.70, -0.26)	-0.70 (-1.48, 0.09)[ns]	-0.71 (-1.49, 0.07)[ns]	-0.93 (-1.84, -0.02)	-0.92 (-1.83, -0.02)	-0.94 (-1.85, -0.04)
**Slow walking**	-1.32 (-2.09, -0.54)	-1.25 (-2.08, -0.41)	-1.10 (-1.94, -0.27)	-1.07 (-2.04, -0.11)	-0.97 (-1.94, 0.00)	-1.40 (-2.37, -0.42)
**Fast walking**	1.35 (0.77, 1.94)	1.24 (0.61, 1.88)	1.13 (0.50, 1.77)	1.24 (0.51, 1.98)	1.12 (0.38, 1.85)	1.15 (0.42, 1.89)
**Combined exercise-like**	-1.01 (-1.26, -0.77)	-0.97 (-1.23, -0.71)	-1.04 (-1.31, -0.78)	-1.11 (-1.41, -0.81)	-1.17 (-1.47, -0.86)	-0.91 (-1.22, -0.60)
**DIASTOLIC BLOOD PRESSURE (no history of CVD)**						
Sample size	n=13184	n=11277		n=8711		
**Sleep**	-1.59 (-2.43, -0.76)	-1.53 (-2.46, -0.61)	-1.54 (-2.46, -0.62)	-1.49 (-2.56, -0.42)	-1.46 (-2.53, -0.39)	-1.44 (-2.50, -0.38)
**Sedentary behavior**	4.34 (3.61, 5.06)	4.15 (3.35, 4.94)	4.22 (3.42, 5.01)	4.32 (3.39, 5.24)	4.36 (3.44, 5.29)	4.48 (3.55, 5.41)
**Standing**	-1.29 (-2.04, -0.53)	-1.17 (-1.99, -0.35)	-1.19 (-2.01, -0.37)	-1.49 (-2.44, -0.53)	-1.47 (-2.43, -0.52)	-1.45 (-2.40, -0.50)
**Slow walking**	-0.81 (-1.61, 0.00) [ns]	-0.72 (-1.58, 0.15) [ns]	-0.58 (-1.45, 0.29) [ns]	-0.52 (-1.53, 0.49) [ns]	-0.44 (-1.45, 0.57) [ns]	-1.23 (-2.24, -0.21)
**Fast walking**	0.78 (0.17, 1.38)	0.61 (-0.04, 1.27) [ns]	0.50 (-0.15, 1.16) [ns]	0.68 (-0.08, 1.44) [ns]	0.56 (-0.20, 1.33) [ns]	0.76 (0.00, 1.52) [ns]
**Combined exercise-like**	-1.43 (-1.68, -1.18)	-1.34 (-1.61, -1.07)	-1.41 (-1.68, -1.14)	-1.50 (-1.81, -1.20)	-1.55 (-1.86, -1.24)	-1.13 (-1.44, -0.81)

[a] Linear regression. Coefficients indicate change in blood pressure per 1 unit increase in the corresponding ILR coordinate. Value >0 indicates more time spent in the behavior relative to others is associated with higher blood pressure; value <0 indicates that more time spent in the behavior relative to others is associated with lower blood pressure. Coefficients indicate the presence of an association, but effect size is not directly interpretable due to the isometric log-ratio transformation.

[b] Restricted to three cohorts: ALSWH, BCS70, TMS

[ns] indicates non-significant results; all other estimates are significant at p<0.05

Table S10. Estimated regression coefficients[a] of isometric log ratios of each behavior (ILR coordinate representing 1 behavior relative to other 6) and **systolic & diastolic blood pressure,** with additional a**djustment for BMI**

**BEHAVIOR**	**Complete cases sample**<br>Model 2: age, sex, cohort, smoking, alcohol, BMI adjusted	**Maximal adjustment sample [b]**<br>Model 3: age, sex, cohort, smoking, alcohol, BMI, education, employment, mobility adjusted
Sample size	n=12623	n=9794
**SYSTOLIC BLOOD PRESSURE**		
**Sleep**	-1.32 (-2.59, -0.05)	-1.24 (-2.69, 0.22) [ns]
**Sedentary behavior**	1.19 (0.06, 2.31)	0.97 (-0.35, 2.28) [ns]
**Standing**	0.40 (-0.75, 1.55) [ns]	0.03 (-1.29, 1.36) [ns]
**Slow walking**	0.59 (-0.63, 1.82) [ns]	0.56 (-0.86, 1.99) [ns]
**Fast walking**	-0.08 (-0.98, 0.82) [ns]	0.33 (-0.72, 1.38) [ns]
**Combined exercise-like**	-0.77 (-1.15, -0.39)	-0.65 (-1.09, -0.21)
**DIASTOLIC BLOOD PRESSURE**		
**Sleep**	-0.41 (-1.23, 0.40)[ns]	-0.27 (-1.21, 0.66)[ns]
**Sedentary behavior**	1.42 (0.69, 2.14)	1.55 (0.70, 2.40)
**Standing**	-1.34 (-2.08, -0.60)	-1.57 (-2.42, -0.71)[ns]
**Slow walking**	0.40 (-0.39, 1.19)[ns]	0.20 (-0.71, 1.12)[ns]
**Fast walking**	0.35 (-0.23, 0.93)[ns]	0.48 (-0.20, 1.15)[ns]
**Combined exercise-like**	-0.41 (-0.66, -0.16)	-0.39 (-0.67, -0.10)

[a] Linear regression. Coefficients indicate change in blood pressure per 1 unit increase in the corresponding ILR coordinate. Value >0 indicates more time spent in the behavior relative to others is associated with higher blood pressure; value <0 indicates that more time spent in the behavior relative to others is associated with lower blood pressure. Coefficients indicate the presence of an association, but effect size is not directly interpretable due to the isometric log-ratio transformation.

[b] Restricted to three cohorts: ALSWH, BCS70, TMS

[ns] indicates non-significant results; all other estimates are significant at p<0.05

Table S11. Estimated regression coefficients[a] of isometric log ratios of each behavior (ILR coordinate representing 1 behavior relative to other 6) and each of **systolic and diastolic blood pressure** stratified by **subgroups of sleep, sedentary behavior and exercise** (based on median cut-points)

	SUBGROUP FOR ANALYSIS					
	**HIGH SLEEP (≥7.2 hours/day)**	**LOW SLEEP (<7.2 hours/day)**	**HIGH SEDENTARY BEHAVIOR (≥10.7 hours/day)**	**LOW SEDENTARY BEHAVIOR (<10.7 hours/day)**	**HIGH EXERCISE (≥10.3min/day)**	**LOW EXERCISE (<10.3 min/day)**
Sample size	n=7381	n=7380	n=7381	n=7380	n=7381	n=7380
**SYSTOLIC BLOOD PRESSURE [b]**						
**Sleep**	**0.72 (-2.02, 3.45) [ns]**	**-3.24 (-5.32,-1.16)**	-3.48 (-5.15,-1.80)	-1.83 (-3.88, 0.23)	-2.16 (-3.98,-0.35)	-3.59 (-5.15,-2.02)
**Sedentary behavior**	**1.66 (-0.49, 3.81) [ns]**	**5.11 (3.54, 6.67)**	5.32 (3.29, 7.35)	2.99 (0.79, 5.18)	4.32 (2.75, 5.89)	4.48 (3.07, 5.90)
**Standing**	-0.28 (-1.83, 1.27) [n]	0.42 (-1.14, 1.98) [ns]	0.03 (-1.62, 1.69) [ns]	0.73 (-0.78, 2.24) [ns]	-0.83 (-2.40, 0.75) [ns]	1.28 (-0.21, 2.77) [ns]
**Slow walking**	-0.34 (-1.98, 1.30) [n]	-1.00 (-2.65, 0.64) [ns]	-0.20 (-1.92, 1.52) [ns]	-0.68 (-2.28, 0.91) [ns]	0.30 (-1.34, 1.94) [ns]	-1.36 (-3.00, 0.27) [ns]
**Fast walking**	0.08 (-1.10, 1.25) [ns]	0.73 (-0.50, 1.97) [ns]	0.22 (-0.97, 1.42) [ns]	0.74 (-0.49, 1.96) [ns]	0.11 (-1.10, 1.33) [ns]	0.61 (-0.61, 1.82) [ns]
**Combined exercise-like**	-1.83 (-2.32,-1.35)	-2.01 (-2.53,-1.50)	-1.90 (-2.39,-1.40)	-1.94 (-2.45,-1.43)	-1.75 (-2.54,-0.96)	-1.42 (-2.16,-0.67)
**DIASTOLIC BLOOD PRESSURE [b]**						
**Sleep**	**2.33 (0.51, 4.15)**	**-2.38 (-3.75,-1.01)**	**-2.55 (-3.66,-1.44)**	**0.24 (-1.12, 1.60) [ns]**	-1.28 (-2.47,-0.09)	-1.81 (-2.86,-0.77)
**Sedentary behavior**	**1.30 (-0.13, 2.73) [ns]**	**4.88 (3.85, 5.90)**	**5.74 (4.40, 7.09)**	**1.70 (0.25, 3.15)**	3.84 (2.81, 4.87)	4.11 (3.16, 5.05)
**Standing**	**-2.36 (-3.39,-1.33)**	**-1.12 (-2.15,-0.09)**	-2.20 (-3.30,-1.11)	-0.72 (-1.71, 0.28) [ns]	**-2.27 (-3.31,-1.24)**	**-0.91 (-1.91, 0.09) [ns]**
**Slow walking**	-0.44 (-1.53, 0.66) [n]	-1.03 (-2.12, 0.05) [ns]	-0.45 (-1.59, 0.69) [ns]	-0.76 (-1.81, 0.30) [ns]	**0.27 (-0.81, 1.35) [ns]**	**-1.41 (-2.50,-0.32)**
**Fast walking**	0.76 (-0.03, 1.54) [ns]	0.80 (-0.02, 1.61) [ns]	0.65 (-0.14, 1.44) [ns]	1.10 (0.29, 1.91)	0.86 (0.06, 1.66)	0.66 (-0.14, 1.47) [ns]
**Combined exercise-like**	**-1.59 (-1.91,-1.27)**	**-1.14 (-1.48,-0.80)**	**-1.19 (-1.51,-0.86)**	**-1.57 (-1.91,-1.23)**	-1.42 (-1.94,-0.90)	-0.63 (-1.13,-0.14)

[a] Linear regression. Coefficients indicate change in blood pressure per 1 unit increase in the corresponding ILR coordinate. Value >0 indicates more time spent in the behavior relative to others is associated with higher blood pressure; value <0 indicates that more time spent in the behavior relative to others is associated with lower blood pressure. Coefficients indicate the presence of an association, but effect size is not directly interpretable due to the isometric log-ratio transformation.

[b] Model adjusted for age, sex, cohort adjusted

[ns] indicates non-significant results; all other estimates are significant at p<0.05

Bolded estimates indicate significant interaction term

Table S12. Characteristics of analytical samples and those excluded due to missing data

	**Maximal analytical sample (n=14761)[1]**		**Complete cases sample[2] (n=12651)**		**Sensitivity analysis sample[3] (n=9799)**		Excluded from all analysis due to missing SBP or DBP data (n=614)		Excluded from complete cases analysis due to missing data (n=2725)		Excluded from sensitivity analysis due to missing data (n=4962)	
	**Mean**	**±SD**										
**24- hour movement behaviors**												
Sleep	428.0	±71.5	426.5	±69.6	428.7	±68.1	454.8	±69.3	441.2	±79.3	426.7	±77.8
Sedentary behavior	642.6	±112	643.5	±110.5	648.3	±107.6	614.0	±111.6	621.9	±118.1	621.2	±118.9
Stand	190.3	±66.1	190.1	±64.9	187.5	±62.2	199.2	±69.0	193.1	±71.8	195.8	±72.8
Slow walk	94.1	±34.5	94.2	±34.1	91.8	±31.8	88.9	±33.2	92.5	±35.9	98.6	±38.9
Fast walk	67.5	±26.9	67.7	±26.6	66.6	±25.4	70.7	±25.8	67.2	±27.8	69.4	±29.5
Combined exercise	16.0	±16.3	16.5	±16.7	15.8	±15.5	9.4	±10.3	12.1	±13.1	16.2	±17.9
**Systolic blood pressure (mmHg)***	132.2	±19.1	133.0	±19.1	132.2	±19.2	-	-	127.8	±18.6	132.2	±19
**Diastolic blood pressure (mmHg)***	79.1	±11.6	79.3	±11.5	78.5	±11.4	-	-	77.8	±12	80.3	±11.9
**Covariates**												
**Age (years)**	54.2	±9.6	54.8	±9.8	55	±9.4	45.3	±3.2	49.3	±6.8	52.6	±9.9
**Mobility limitations (0-100 score)**	87.1	±18.9	87.7	±17.9	88.3	±17.2	90.1	±15.0	85.1	±22.1	83.7	±22.9
	**N**	**(%)**										
**Sex** Female	7828	(53.0)	6632	(52.4)	5091	(52.0)	591	(96.3)	1787	(65.6)	2737	(55.2)
Male	6933	(47.0)	6019	(47.6)	4708	(48.0)	23	(3.7)	937	(34.4)	2225	(44.8)
**Cohort** TMS	7509	(50.9)	6883	(54.4)	6079	(62.0)	6	(1.0)	632	(23.2)	1430	(28.8)
ALSWH	425	(2.9)	425	(3.4)	425	(4.3)	560	(91.1)	560	(20.6)	0	(0)
BCS70	5212	(35.3)	3819	(30.2)	3295	(33.6)	38	(6.2)	1431	(52.5)	1917	(38.6)
DPhacto	830	(5.6)	780	(6.2)	-	-	5	(0.8)	55	(2.0)	830	(16.7)
FIREA	248	(1.7)	241	(1.9)	-	-	6	(1.0)	13	(0.5)	248	(5.0)
NES	537	(3.6)	503	(4.0)	-	-	0	(0)	34	(1.2)	537	(10.8)
**Smoking** Non-smoker	12491	(85.1)	10884	(86.0)	8530	(87.0)	564	(92.0)	2171	(82.4)	3961	(81.3)
Smoker	2181	(14.9)	1767	(14.0)	1269	(13.0)	49	(8.0)	463	(17.6)	912	(18.7)

**Alcohol**	Tertile 1 (low)	4298	(33.8)	4267	(33.7)	3247	(33.1)	205	(34.1)	236	(35.2)	1051	(36.0)
	Tertile 2	4332	(34.1)	4308	(34.1)	3357	(34.3)	210	(34.9)	234	(34.9)	975	(33.4)
	Tertile 3 (high)	4090	(32.2)	4076	(32.2)	3195	(32.6)	187	(31.1)	201	(30.0)	895	(30.6)
**Education**	None or less than high school	1658	(12.3)	1186	(10.3)	990	(10.1)	11	(1.8)	483	(18.8)	668	(18.0)
	High school (~16y)	3879	(28.7)	3242	(28.1)	2749	(28.1)	36	(6.0)	673	(26.1)	1130	(30.5)
	Further education (~16-18y)	5191	(38.4)	4704	(40.8)	4098	(41.8)	235	(39.0)	722	(28.0)	1093	(29.5)
	University degree or higher	2781	(20.6)	2404	(20.8)	1962	(20.0)	320	(53.2)	697	(27.1)	819	(22.1)
**Occupational class**	Not working	3786	(29.9)	3558	(32.3)	3505	(35.8)	70	(11.6)	298	(13.2)	281	(9.8)
	Low	2168	(17.1)	1746	(15.9)	1067	(10.9)	38	(6.3)	460	(20.4)	1101	(38.4)
	Intermediate	3517	(27.8)	2936	(26.7)	2649	(27.0)	158	(26.1)	739	(32.7)	868	(30.3)
	High	3192	(25.2)	2770	(25.2)	2578	(26.3)	339	(56.0)	761	(33.7)	614	(21.4)

[1] Individuals have information on SBP, DBP, age, sex, cohort

[2] Individuals have complete data on SBP, DBP, age, sex, cohort, smoking status, alcohol consumption

[3] Individuals have complete data on SBP, DBP, age, sex, cohort, smoking status, alcohol consumption, employment, education, mobility limitations

*=+10mmHg if individuals are currently on anti-hypertensive medications

ALSWH Australian Longitudinal Study of Women's Health; **BCS70** 1970 British Cohort Study; **DPhacto** Danish PHysical ACTivity cohort with Objective measurements; **FIREA** Finnish Retirement and Aging Study; **NES** Nijmegen Exercise Study; **SD** standard deviation; **TMS** The Maastricht Study

Figure S1. Derivation of analytical sample size. See cohort profiles for more information on rates of participation in accelerometer data collection and reasons for missing data

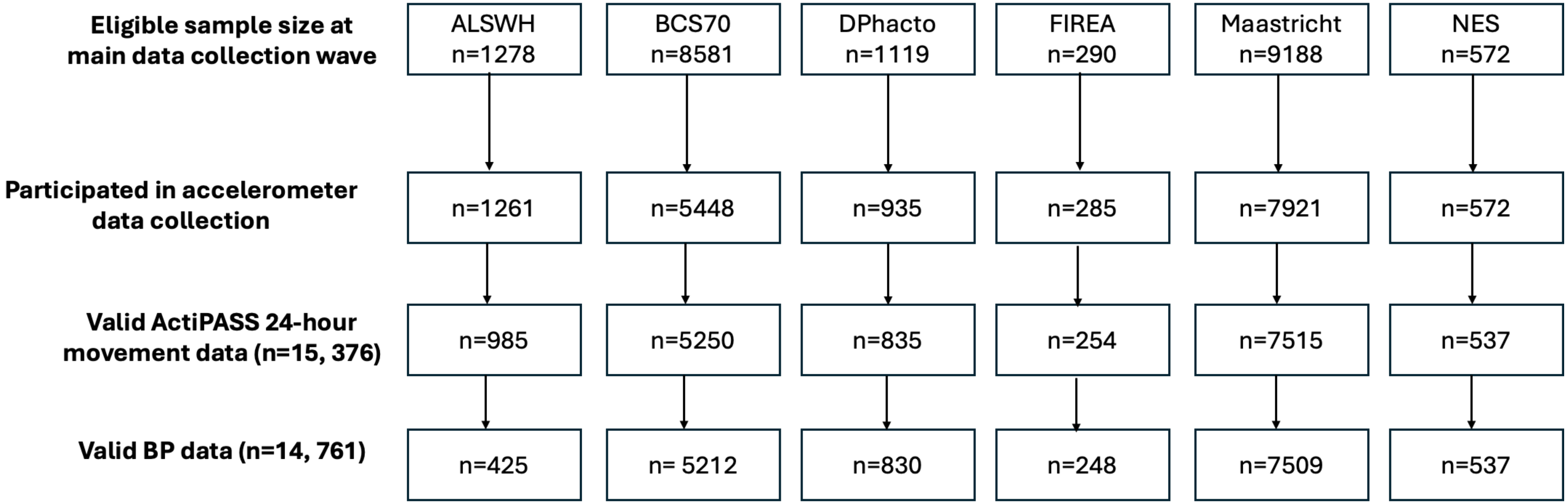

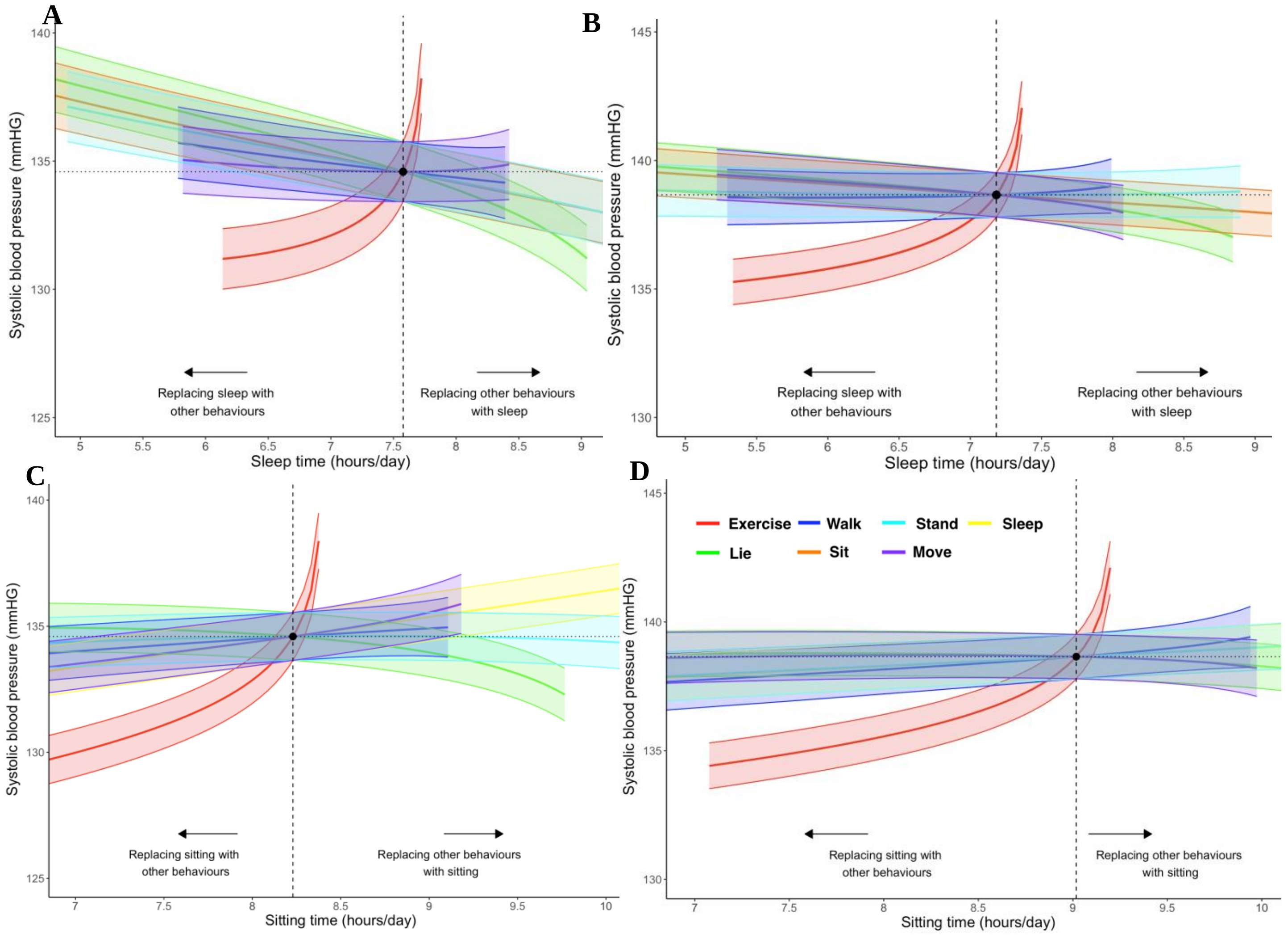

Figure S2. Theoretical change in **systolic** blood pressure (n=) based on behavioral relocation for (*A*) sleep in females; (*B*) sleep in males; (C) sitting in females; (D) sitting in males. Data to the left of the reference line indicate the predicted change in systolic blood pressures if a given behavior is replaced by any of the other behaviors. Data to the right of the reference line indicate the predicted change if a given behavior replaces any of the other behaviors. Model adjusted for age (ref: 54.2 years; mean-centred), and cohort (ref: Maastricht Study).

Figure S3. Forest plot of two-stage meta-analysis demonstrating estimates by cohort for **<u>systolic blood pressure</u>**

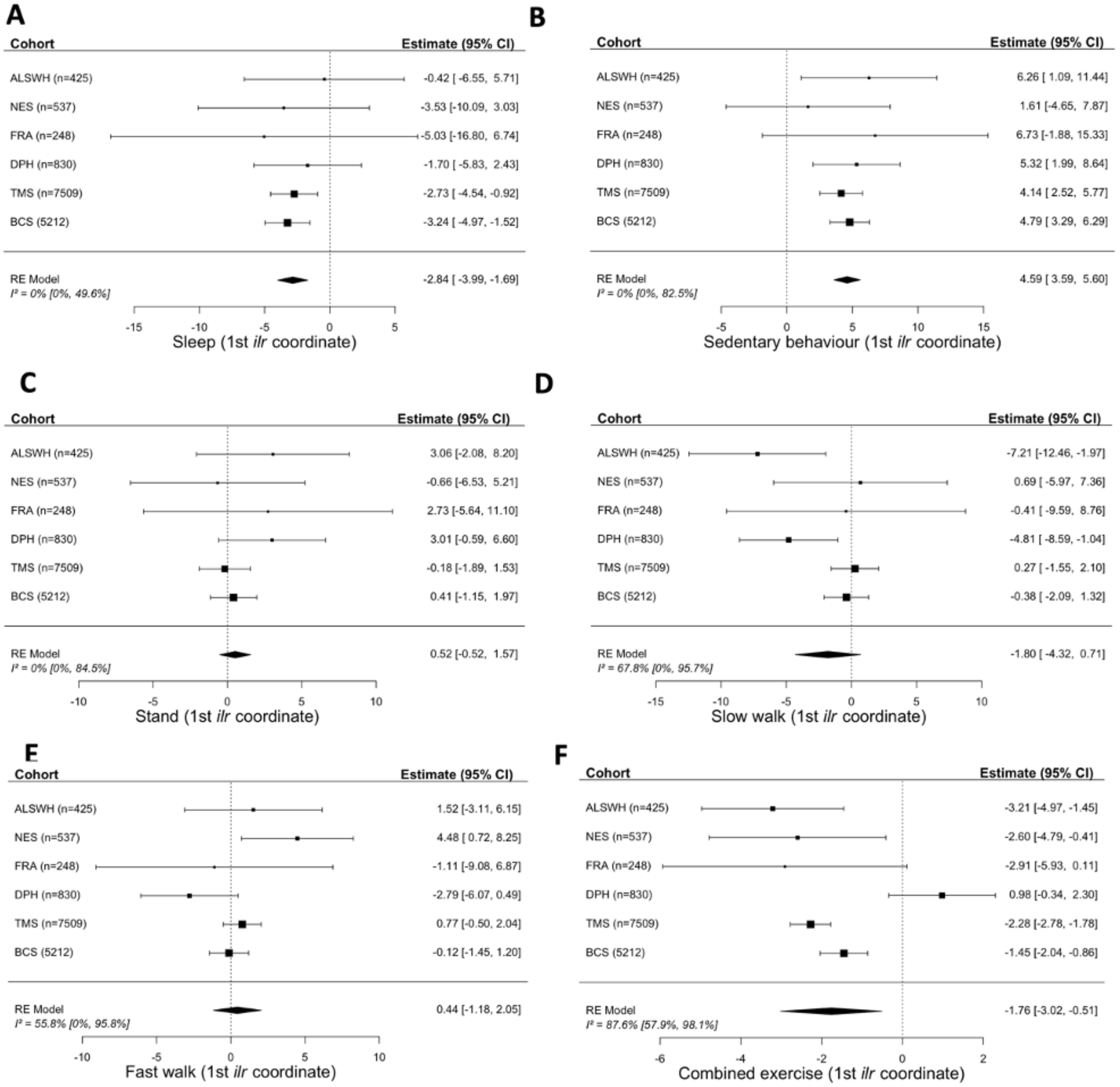

Figure S4. Forest plot of two-stage meta-analysis demonstrating estimates by cohort for **diastolic blood pressure**

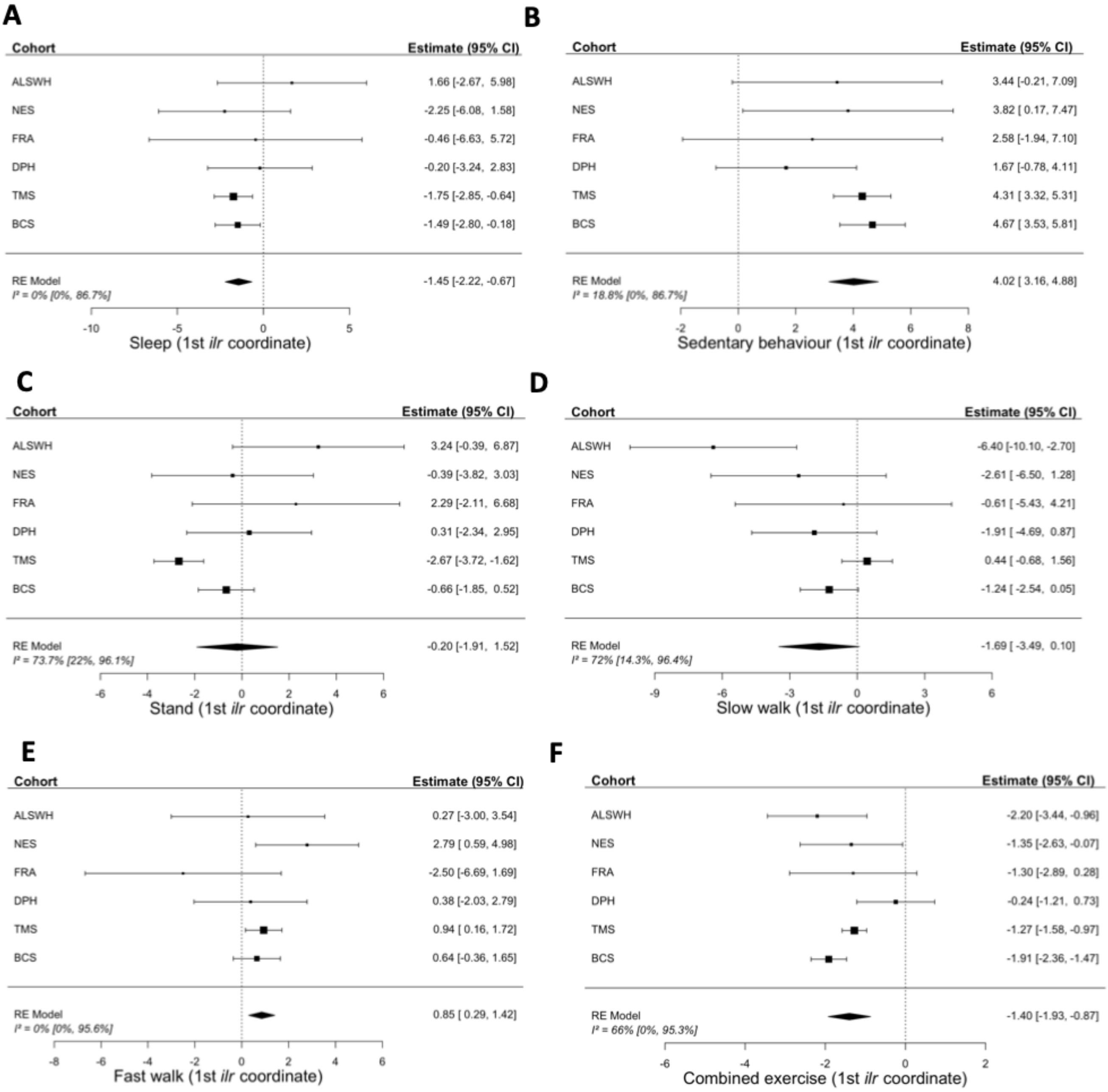

Supplement: Supplementary file 1 [file cir-151-159-s001.pdf]
